# Multi sequence average templates for aging and neurodegenerative disease populations

**DOI:** 10.1038/s41597-022-01341-2

**Published:** 2022-05-27

**Authors:** Mahsa Dadar, Richard Camicioli, Simon Duchesne

**Affiliations:** 1 Department of Psychiatry, Faculty of Medicine, McGill University, Montreal, QC Canada; 2 Department of Medicine, Division of Neurology, University of Alberta, Edmonton, AB Canada; 3 Department of Radiology and Nuclear Medicine, Faculty of Medicine, Laval University, Quebec, QC Canada

**Keywords:** Neurodegenerative diseases, Neurological disorders, Medical research

## Abstract

Magnetic resonance image (MRI) processing pipelines use average templates to enable standardization of individual MRIs in a common space. MNI-ICBM152 is currently used as the standard template by most MRI processing tools. However, MNI-ICBM152 represents an average of 152 healthy young adult brains and is vastly different from brains of patients with neurodegenerative diseases. In those populations, extensive atrophy might cause inevitable registration errors when using an average template of young healthy individuals for standardization. Disease-specific templates that represent the anatomical characteristics of the populations can reduce such errors and improve downstream driven estimates. We present multi-sequence average templates for Alzheimer’s Dementia (AD), Fronto-temporal Dementia (FTD), Lewy Body Dementia (LBD), Mild Cognitive Impairment (MCI), cognitively intact and impaired Parkinson’s Disease patients (PD-CIE and PD-CI, respectively), individuals with Subjective Cognitive Impairment (SCI), AD with vascular contribution (V-AD), Vascular Mild Cognitive Impairment (V-MCI), Cognitively Intact Elderly (CIE) individuals, and a human phantom. We also provide separate templates for males and females to allow better representation of the diseases in each sex group.

## Abstract (summary)

**Table:** 

Measurement(s)	Human Brain
Technology Type(s)	Magnetic resonance imaging
Sample Characteristic - Organism	Homo Sapiens
Sample Characteristic - Location	Canada

## Background & Summary

Magnetic resonance imaging (MRI) brain templates (i.e. averages of multi-individual images, co-registered in a similar reference space) are widely used in image processing, for example as targets in registration and intensity normalization, as a common standard space enabling individual and population based comparisons in deformation/tensor or voxel based morphometry, and as the basis for segmentation techniques that rely on nonlinear registration^1–4^. An example is the MNI-ICBM152, an average based on images from 152 healthy young adults, and one of the most popular templates in current use given its distribution in processing pipelines such as MINC, FSL, and SPM^1–3^ that have been shared more than 45,000 times worldwide (Data from NITRC.org).

A common feature of existing averages such as the MNI-ICBM152 is their reliance on healthy, young brains, in addition to aggregating both sexes in the template generation process. However, in aging and populations with neurodegenerative diseases, ventricle enlargement, extensive levels of cortical and subcortical atrophy, as well as white matter hyperintensities (WMHs) create large degrees of difference between an individual’s MRI and such templates. We have shown in prior work that such differences significantly increase registration errors in some of these well-known image processing tools (e.g. ANTs, Elastix, FSL, MINC, and SPM)^5^. Ridwan *et al*. have shown that use of age-appropriate templates allows for higher inter-subject spatial normalization accuracy for older adult data, facilitating detection of smaller inter-group morphometric differences^6^. A similar reasoning applies to studies of neurodegeneration. Using a dataset consisting of patients with different frontotemporal dementia variants, we have shown that use of age and disease appropriate templates can significantly reduce nonlinear registration errors^7^. Van Hecke *et al*. have also shown that improvement in image alignments due to use of population-specific atlases leads to higher sensitivity and specificity in detecting white matter abnormalities in diffusion tensor imaging (DTI) voxel-based analyses^8^. Therefore, age and disease appropriate templates are necessary to reflect the anatomical characteristics of the populations of interest and increase downstream accuracy and sensitivity of the analyses by reducing potential image processing errors and biases that can occur when using age and pathology inappropriate templates^7^. An example use case would be the monitoring of a therapy in a specific pathology, with an effect that may be clinically significant but resulting in small image differences. The increased sensitivity brought about by using an appropriate, age-, sex- and disease template would therefore be significant.

Previous work on average brain templates has been mostly based on pediatric, young adult, or healthy aged brains^6,9–13^. Xiao *et al*. have developed a multi-contrast template of 15 Parkinson’s disease patients^14^. We have previously developed average T1w templates of frontotemporal dementia variants (i.e. behavioural, semantic, and progressive non-fluent aphasia) along with age matched healthy templates, showing that use of age and disease appropriate templates improve nonlinear registration performance^7^. Guo *et al*. have recently developed a T1w brain template based on a combination of healthy aged adults, individuals with mild cognitive impairment, and Alzheimer’s disease patients, showing that use of disease-specific templates improves sensitivity in voxel-based gray matter volume analyses, enabling for early detection and earlier therapeutic opportunities^15^. To our knowledge, no prior work has provided multi-sequence average templates of various neurodegenerative disease populations generated consistently using harmonized image acquisition protocols.

Based on data from the Canadian Consortium for Neurodegeneration and Aging (CCNA)^16^, a flagship study of the Canadian Institutes of Health Research, we present average templates for T1-weighted (T1w), T2-weighted (T2w), T2*-weighted, Proton Density (PD), and FLuid Attenuated Inversion Recovery (FLAIR) sequences in eleven diagnostic groups, including Alzheimer’s Dementia (AD), Fronto-temporal Dementia (FTD), Lewy Body Dementia (LBD), Mild Cognitive Impairment (MCI), cognitively intact and impaired Parkinson’s Disease patients (PD-CIE and PD-CI, respectively), individuals with Subjective Cognitive Impairment (SCI), Vascular Alzheimer’s Dementia (V-AD), Vascular Mild Cognitive Impairment (V-MCI), as well as Cognitively Intact Elderly (CIE) individuals and one human phantom^17^. These templates can capture the anatomical characteristics for each disease cohort at the regional level. With multiple contrasts available providing different types of information, the various templates can be used to assess different aspects in each disease: i) T1w templates are useful for assessing fine anatomical details and estimating regional and global atrophy levels; ii) T2w/PD sequences are useful for skull segmentation, and assessment of deep gray matter structures, iii) FLAIR images can be used to detect WMHs and infarcts; and iv) T2* images can be used to identify microbleeds as well as hemorrhages.

There are significant sex and gender related differences in the prevalence, clinical outcomes, and response to treatments for these distinct neurodegenerative diseases (e.g. higher prevalence of Alzheimer’s disease in females and higher prevalence of Parkinson’s disease in males)^18–20^. Sex-specific average templates would therefore be useful tools to represent and assess potential anatomical differences in patterns of atrophy in males and females. Thus, in addition to the disease-specific average templates combining male and female participants, we provide separate templates for males and females in each diagnostic category.

## Methods

### Data

We used data from the Comprehensive Assessment of Neurodegeneration and Dementia (COMPASS-ND) cohort of the CCNA, a national initiative to catalyze research on dementia^16^. COMPASS-ND includes deeply phenotyped subjects with various forms of dementia and mild memory loss or concerns, along with cognitively intact elderly subjects. Ethical agreements were obtained at all respective sites. Written informed consent was obtained from all participants.

Clinical diagnoses were determined by participating clinicians based on longitudinal clinical, screening, and MRI findings (i.e. diagnosis reappraisal was performed using information from recruitment assessment, screening visit, clinical visit with physician input, and MRI). The diagnostic groups included, AD, CIE, FTD, LBD, MCI, PD-CIE, PD-MCI, PD-Dementia (for this study, PD-MCI and PD-Dementia groups were merged into one PD-CI group), SCI, V-AD, and V-MCI. Diagnosis was performed according to the current guidelines in the field and diagnostic criteria was harmonized across all CCNA sites. However, we acknowledge that due to the inherent heterogeneity and variabilities in such neurodegenerative disease populations, there might be inevitable variabilities across different centers and studies. For details on clinical group ascertainment, see Pieruccini‐Faria *et al*.^21^ as well as Dadar *et al*.^22^ (section 1 in the supplementary materials). A single cognitively healthy volunteer was also scanned as a human phantom multiple times across different centers for quality assurance purposes (more information on the SIMON human phantom dataset can be found in Duchesne *et al*.^17^).

Table 1 summarizes the demographic characteristics of the participants used to generate each template. Note that due to the lower prevalence and challenges in recruitment of participants in certain disease categories (e.g. FTD and LBD), the resulting templates might not be reflective of the entire spectrum of presentation of the pathology. Further work including larger populations is therefore warranted.

**Table 1:** Demographic characteristics of the participants used to create the average templates.

Measure	N			Age			P Value
Diagnosis	Total	Female	Male	Total	Female	Male	
AD	73	29	44	74.34 ± 7.58	73.09 ± 7.57	75.17 ± 7.55	0.25
CIE	94	76	18	70.18 ± 6.05	70.21 ± 6.03	70. 03 ± 6.33	0.91
FTD	28	16	12	66.91 ± 8.29	65.95 ± 6.77	68.20 ± 10.15	0.49
LBD	21	2	19	72.25 ± 8.11	73.68 ± 2.52	72.10 ± 8.51	0.80
MCI	210	92	118	72.04 ± 6.66	71.43 ± 6.66	72.51 ± 6.65	0.24
Mixed	41	22	19	78.89 ± 6.63	80.45 ± 6.69	77.26 ± 6.32	0.12
PD-CIE	65	31	34	66.66 ± 6.91	67.79 ± 6.38	65.69 ± 7.28	0.22
PD-CI	45	7	38	72.01 ± 7.58	67.84 ± 13.01	72.75 ± 6.13	0.12
SCI	125	93	32	70.57 ± 5.91	70.92 ± 5.95	69.58 ± 5.75	0.27
V-AD	27	11	16	77.34 ± 7.07	76.47 ± 6.65	78.05 ± 7.53	0.56
V-MCI	135	61	74	76.22 ± 6.32	74.32 ± 6.21	77.78 ± 6.01	0.001
SIMON	68	—	68	44.75 ± 1.47	—	44.75 ± 1.47	—

All participants were scanned using the Canadian Dementia Imaging Protocol, a harmonized MRI protocol designed to reduce inter-scanner variability in multi-centric studies and which included the following sequences^23^:

- 3D isotropic T1w scans (voxel size = 1.0 × 1.0 × 1.0 mm^3^) with an acceleration factor of 2 (Siemens: MP‐RAGE‐PAT: 2; GE: IR‐FSPGR‐ASSET 1.5; Philips: TFE‐Sense: 2)
- Interleaved proton density/T2‐weighted (PD/T2w) images (voxel size = 0.9 × 0.9 × 3 mm^3^), fat saturation, and an acceleration factor of 2.
- Fluid attenuated inversion recovery (T2w‐FLAIR) images (voxel size = 0.9 × 0.9 × 3 mm^3^), fat saturation, and an acceleration factor of 2.
- T2* gradient echo images (voxel size = 0.9 × 0.9 × 3 mm^3^) and acceleration factor of 2.

Table 2 shows the acquisition parameters for each sequence and scanner manufacturer. A detailed description, exam cards, and operators’ manual are publicly available at: www.cdip-pcid.ca.

**Table 2:** Acquisition parameters of the CDIP protocol.

Sequence	Scanner Model	Matrix	Resolution (mm^3^)	Number of Slices	TR (msec)	TE (msec)	TI (msec)	Flip Angle
T1w	GE	256 × 256	1.0 × 1.0 × 1.0	180	6.7	2.9	400	11
	Philips	256 × 248	1.0 × 1.0 × 1.0	180	7.3	3.3	935	9
	Siemens	256 × 256	1.0 × 1.0 × 1.0	192	2300	2.98	—	9
T2w/PD	GE	256 × 256	0.94 × 0.94 × 3.0	48	3000	11/85	—	125
	Philips	256 × 254	0.94 × 0.94 × 3.0	48	3000	13/100	—	90
	Siemens	256 × 256	0.94 × 0.94 × 3.0	48	3000	10/91	—	165
FLAIR	GE	256 × 256	0.94 × 0.94 × 3.0	48	9000	140	2500	125
	Philips	256 × 224	0.94 × 0.94 × 3.0	48	9000	125	2500	150
	Siemens	256 × 256	0.94 × 0.94 × 3.0	48	9000	123	2500	165
T2*	GE	256 × 256	0.94 × 0.94 × 3.0	48	650	20	—	20
	Philips	256 × 256	0.94 × 0.94 × 3.0	48	650	20	—	20
	Siemens	256 × 256	0.94 × 0.94 × 3.0	48	650	20	—	20

TR: repetition time; TE: echo time; TI: inversion time.

### Preprocessing

All images were pre-processed with image denoising^24^, intensity non-uniformity correction^25^, and image intensity normalization into a 0–100 range. The pre-processed images were then linearly^5^ registered to the pseudo-Talairach space defined by the MNI-ICBM152-2009c template using a 9-parameter registration (three translation, three rotation, and three scaling parameters)^26^. T2w, PD, FLAIR, and T2* images were also co-registered (rigid registration, 6 parameters) to the T1w images with a mutual information cost function.

### Template generation

The method by Fonov *et al*. was used to generate unbiased templates for each diagnostic group for all participants, as well as each group but separately for males and females^12,27^ (all except the LBD group in which there were only two female participants). This method has previously been used to generate templates in various studies, including the latest higher resolution version of the MNI-ICBM2009c template (http://nist.mni.mcgill.ca/?p=904)^26,28^. In short, the pipeline implements a hierarchical nonlinear registration procedure using Automatic Nonlinear Image Matching and Anatomical Labelling (ANIMAL)^29^, iteratively refining the previous registrations by reducing the step size (20 iterations in total, four iterations at each of the levels of 32, 16, 8, 4, and 2 mm, respectively) until convergence is reached. This process of increasingly refined iterative nonlinear registrations leads to average brains that reflect the anatomical characteristics of the population of interest with higher levels of anatomical detail^27^. The higher resolution T1w images (isotropic 1mm^3^) were used to obtain the nonlinear transformations for creating the average templates. T2w, PD, FLAIR, and T2* templates were then created by combining their rigid to-T1w co-registration transformations with the nonlinear transformations based on the T1w images. All final templates were generated at 1mm^3^ isotropic resolution.

### FreeSurfer segmentation

To appreciate differences between templates, we processed all T1w averages using *FreeSurfer* version 6.0.0 (*recon-all -all*). *FreeSurfer* provides a full processing stream for structural T1w data (https://surfer.nmr.mgh.harvard.edu/)^30^. The final segmentation output (aseg.mgz) was then used to obtain volumetric information for each template based on the FreeSurfer look up table available at https://surfer.nmr.mgh.harvard.edu/fswiki/FsTutorial/AnatomicalROI/FreeSurferColorLUT.

## Data Records

For information on COMPASS-ND dataset and to request access, see https://ccna-ccnv.ca/compass-nd-study/. The average template files for all groups and sequences are available in both compressed MINC^31,32^ and NIfTI formats at G-Node (https://gin.g-node.org/mahsadadar/CDIP_Templates)^33^ as well as Zenodo ^34^.

## Technical Validation

### Quality control

**Fig. 1:**
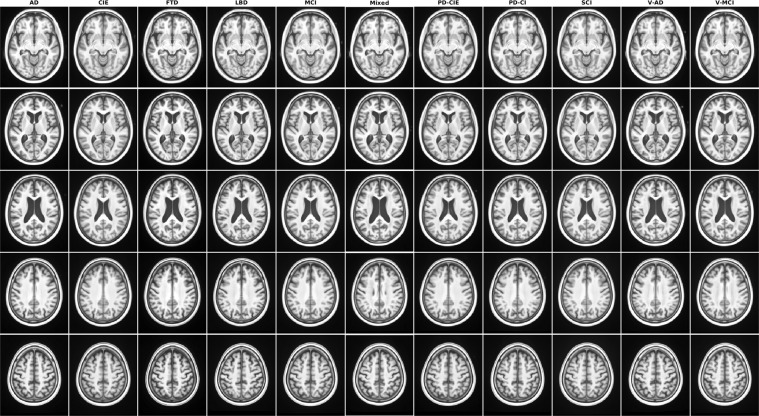
Axial slices of T1w average templates for all diagnostic groups.

The quality of the registrations, pre-processed images, as well as the volumetric segmentations performed by FreeSurfer was visually assessed by an experience rater (MD). All images passed this quality control step. Note that the provided data was already quality controlled by the CCNA imaging platform for presence of imaging artifacts, and only scans that had passed this quality control step were acquired and used for this study. In terms of qualitative comparison with other atlases in the field^6,9,10,14,27^, based on visual assessment, the provided atlases have high levels of image sharpness and anatomical detail, clearly delineating the sulci and gyri in the cortex (Fig. 1).

### Templates

**Fig. 2:**
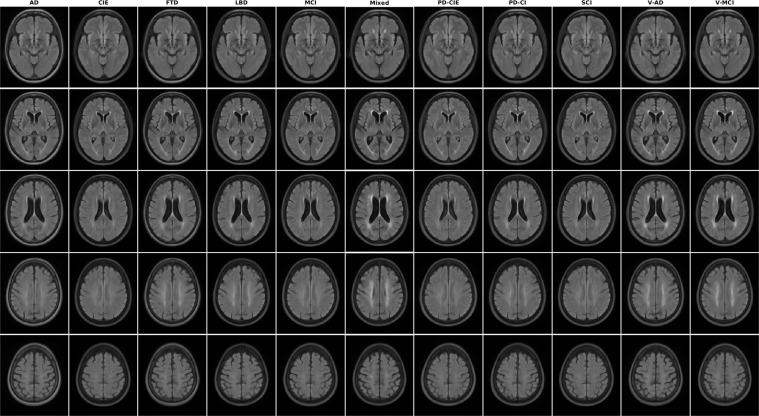
Axial slices of FLAIR average templates for all diagnostic groups.

**Fig. 3:**
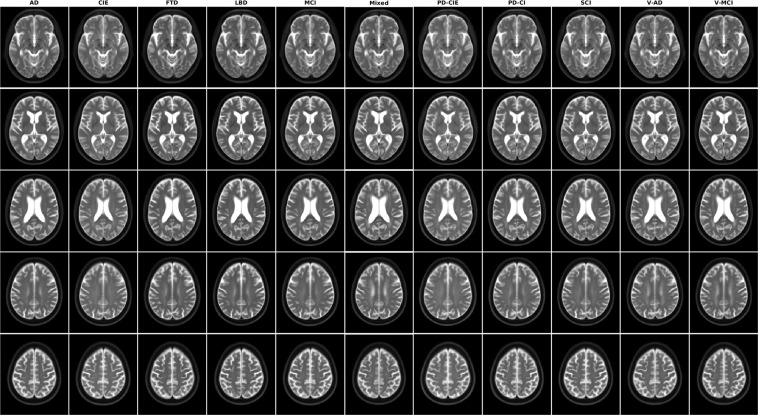
Axial slices of T2w average templates for all diagnostic groups.

**Fig. 4:**
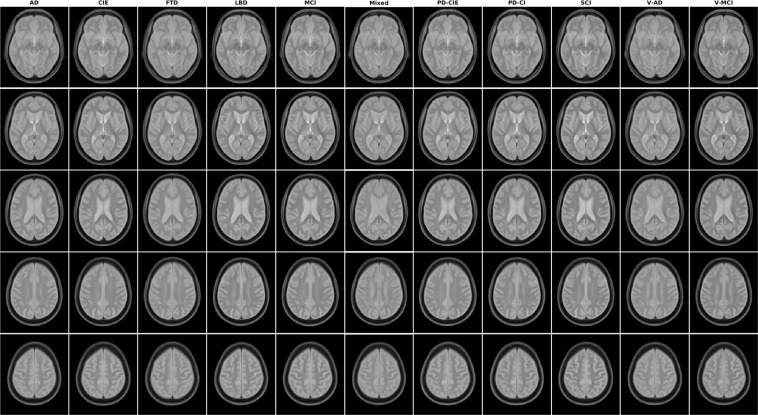
Axial slices of PD average templates for all diagnostic groups.

**Fig. 5:**
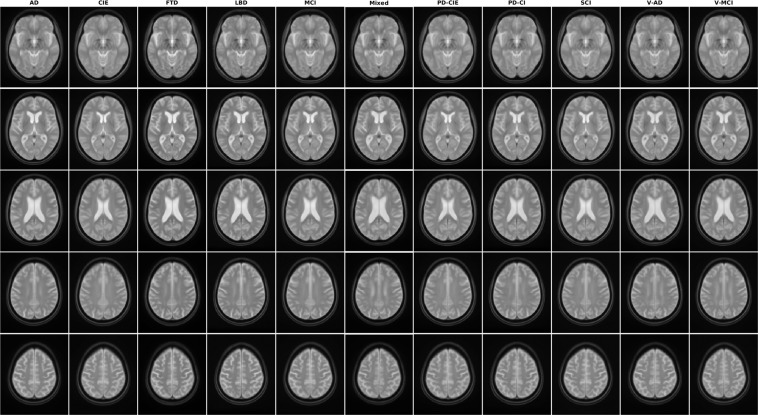
Axial slices of T2* average templates for all diagnostic groups.

Figures 1–5 show axial slices of the T1w, T2w, T2star, PD, and FLAIR average templates for all 11 diagnostic groups, covering the brain at different levels. For more detailed figures of each template, see the supplementary materials (Figures S1–S11). As expected, CIE, PD-CIE, and MCI groups had smaller ventricles, with lower levels of atrophy compared with the cognitively impaired and dementia groups (Fig. 1). FLAIR images of the vascular cohorts (i.e. Mixed, V-MCI, and V-AD) showed extensive levels of periventricular hyperintensities compared to other groups (Fig. 2), due to the presence of WMHs in the majority of the patients in these populations. This pattern was also visible to a lesser extent as hypointensity in the T1w templates, as well as hyperintensity in the T2w, PD, and T2* templates (Figs. 3 to 5). Presence of WMHs is another factor that necessitates use of age and disease appropriate templates, since they can directly impact intensity normalization results. In fact, we have previously shown that presence of WMHs significantly reduces linear registration accuracy in currently used image processing pipelines such as MINC, FSL, Elastix, SPM, and ANTs when images with high WMH burden are registered to young and healthy adult templates such as MNI-ICBM152^5^. Similarly, we showed that increased ventricular volume due to aging and presence of atrophy (e.g. in AD populations) reduces registration accuracy when using healthy young adult templates as the registration target^5^.

Figure 6 shows axial slices of the male and female templates for all diagnostic groups and sequences. Overall, male templates have larger ventricles and greater levels of atrophy than female templates. For more detailed figures of each template, see the supplementary materials (Figures S12–S31).

**Fig. 6:**
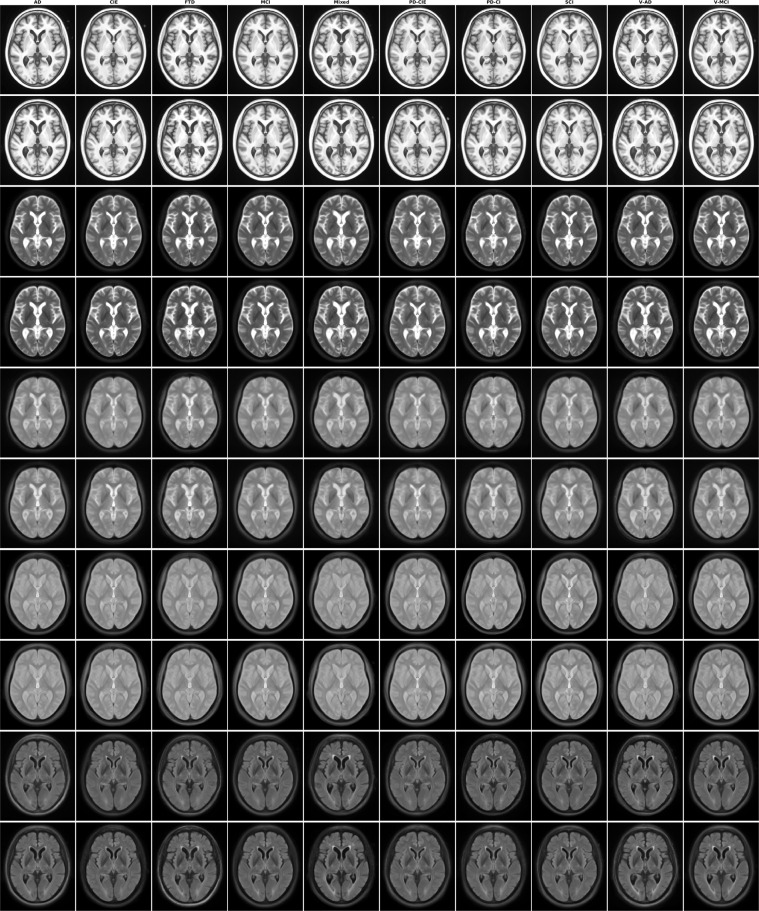
Axial slices of average male and female templates for all sequences and diagnostic groups.

**Fig. 7:**
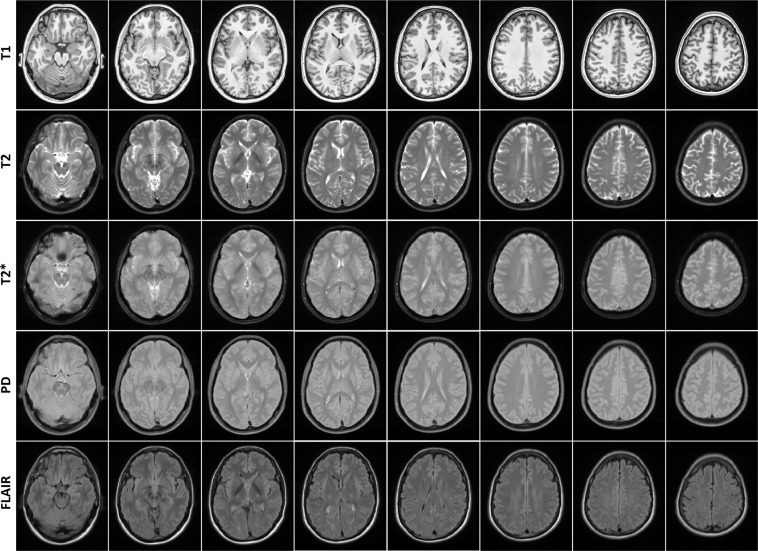
Axial slices of human phantom (SIMON) templates for all sequences.

Figure 7 shows axial slices of the templates for the human phantom (SIMON).

### Volumetric comparisons

**Fig. 8:**
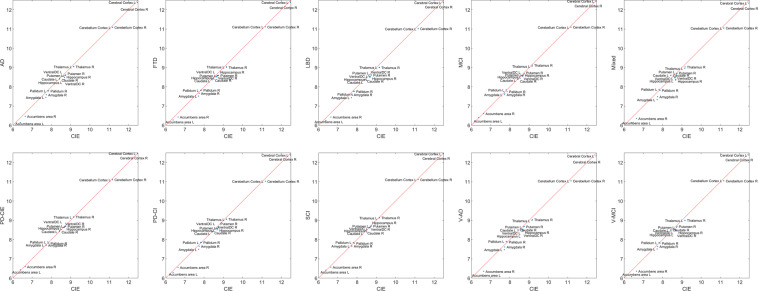
FreeSurfer based GM volumes for each diagnostic group versus the CIE template. CIE = Cognitively Intact Elderly. L: Left. R: Right.

Tables 3–5 summarize the grey and white matter (GM, WM) and cerebrospinal fluid (CSF) volumetric information for the templates as segmented by FreeSurfer. Figure 8 compares GM volumes (log transformed) of each template against the CIE template. Data points below the reference line (shown in red) indicate lower values for the template in comparison with the CIE template. As expected, cognitively impaired and dementia templates had lower GM values than the CIE template, whereas both cognitively intact PD-CIE and SCI templates had similar volumes to the CIE template (i.e. data points fall on the reference line).

**Table 3:** Volumetric GM information (in mm^3^) for each template based on FreeSurfer segmentations.

Region	Template	AD	CIE	FTD	LBD	MCI	Mixed	PD-CIE	PD-CI	SCI	V-AD	V-MCI
Left Cerebral Cortex	All	237797	262462	240082	235997	255191	231215	260165	238048	256237	229139	238411
	Female	246512	272764	243996	—	259307	234007	265552	257829	263501	239656	248613
	Male	233287	252588	230749	—	250642	225176	255056	236250	250396	220836	230674
Left Cerebellum Cortex	All	64885	71013	66955	60818	65282	62459	66035	61471	67262	62735	63170
	Female	69322	71422	69872	—	68839	65264	67637	67170	67356	66924	65645
	Male	63609	69509	64568	—	63486	61363	63448	60585	67575	60464	60554
Left Thalamus Proper	All	8500	9406	8430	8211	8651	7774	9535	8505	9147	7965	7996
	Female	8783	9593	8672	—	8986	7959	9760	9813	8838	8646	8449
	Male	8226	9293	7781	—	8702	7581	9131	8532	9044	7779	7582
Left Caudate	All	4453	4510	4307	4298	4378	5424	4532	4407	4641	4896	4849
	Female	4670	4594	4158	—	4509	6344	4637	4828	4781	5034	5023
	Male	4194	4522	4812	—	4280	5119	4370	4219	4449	4813	5011
Left Putamen	All	5391	6120	5345	5557	5592	5775	6000	5472	6015	5779	5618
	Female	5535	5909	5398	—	5845	6266	5993	6250	6037	5540	5706
	Male	5182	6112	5183	—	5494	5536	5851	5618	5739	5730	5742
Left Pallidum	All	2419	2473	2487	2365	2547	2624	2624	2572	2561	2712	2537
	Female	2495	2562	2500	—	2665	2403	2721	2617	2603	2539	2655
	Male	2472	2504	2567	—	2465	2664	2567	2489	2514	2600	2658
Left Hippocampus	All	4341	5821	5271	4864	5142	4239	5600	4939	5495	4588	4775
	Female	4785	5893	5649	—	5535	4579	5591	5461	5538	5105	5036
	Male	4346	5654	4688	—	5157	4123	5222	4963	5309	4593	4626
Left Amygdala	All	1731	2235	1845	1699	1986	1531	2240	1788	2120	1760	1824
	Female	1643	2178	1811	—	2048	1445	2215	1917	2164	1790	1678
	Male	1656	2169	1604	—	2062	1414	2208	1903	1960	1710	1686
Left Accumbens area	All	465	594	533	523	608	460	618	538	609	390	434
	Female	634	596	518	—	552	484	627	602	592	500	513
	Male	468	607	529	—	563	429	608	572	591	448	485
Left Ventral DC	All	5267	5768	4967	5157	5328	4794	5702	5389	5565	5097	5109
	Female	5620	5733	5272	—	5668	4995	5553	5593	5583	5605	5185
	Male	5034	5411	4783	—	5485	4843	5737	5499	5745	4802	4997
Right Cerebral Cortex	All	239466	260322	242761	236041	252394	229193	259212	240757	258219	228990	240255
	Female	248986	272888	246674	—	260026	236469	266584	259740	264036	243459	250426
	Male	237412	255687	235875	—	249127	228681	250634	236027	251961	219618	230694
Right Cerebellum Cortex	All	65439	71032	66909	61442	66182	63352	67076	61595	67253	62888	62742
	Female	68914	71521	70325	—	68931	64300	68501	67249	67507	66664	65893
	Male	64702	69301	63763	—	63706	61902	65100	60928	68573	60207	60918
Right Thalamus Proper	All	8457	9538	8220	8117	9156	8364	9702	8614	9300	8395	8072
	Female	8893	9503	8849	—	9424	8255	9832	9981	9040	8749	8650
	Male	8086	8933	7697	—	8552	7933	9224	8551	9346	7979	8080
Right Caudate	All	4662	4586	4598	4301	4504	5430	4765	4555	4706	4934	4958
	Female	5012	4664	4462	—	4953	5806	4847	4626	4852	5052	5087
	Male	4364	4863	4738	—	4233	5583	4484	4366	4623	5095	4841
Right Putamen	All	5538	6286	5414	5506	5921	6027	5883	5739	6011	5851	5903
	Female	5842	6080	5454	—	6020	5891	6164	6320	6155	5906	6164
	Male	5528	6137	5372	—	5927	5820	5863	5904	5877	5831	5846
Right Pallidum	All	2410	2583	2510	2363	2318	2476	2469	2563	2460	2618	2469
	Female	2617	2655	2338	—	2636	2442	2553	2555	2662	2597	2510
	Male	2409	2407	2372	—	2347	2401	2634	2537	2391	2565	2430
Right Hippocampus	All	4825	5903	5395	5110	5437	4405	5809	5212	5741	4821	4881
	Female	5055	5941	5670	—	5615	4714	5910	5709	5670	5165	5201
	Male	4632	5781	5013	—	5377	4338	5540	5224	5505	4715	4775
Right Amygdala	All	1979	2295	2135	1997	2147	1782	2230	2106	2133	2027	2082
	Female	1944	2330	2116	—	2246	1682	2295	2095	2139	1944	1944
	Male	1924	2404	1899	—	2246	1743	2208	2215	2255	1876	1853
Right Accumbensarea	All	648	768	623	622	744	575	726	704	709	574	576
	Female	750	726	654	—	708	679	776	774	696	662	638
	Male	589	697	632	—	655	562	751	689	701	620	641
Right Ventral DC	All	5176	5513	5100	5244	5318	4788	5575	5299	5460	5097	5054
	Female	5418	5434	5262	—	5623	5173	5429	5632	5381	5216	5141
	Male	5088	5334	4852	—	5382	4876	5331	5293	5650	4892	5005

**Table 4:** Volumetric WM information (in mm^3^) for each template based on FreeSurfer segmentations.

Region	Template	AD	CIE	FTD	LBD	MCI	Mixed	PD-CIE	PD-CI	SCI	V-AD	V-MCI
Left Cerebrum	All	291772	307530	288626	298345	309407	293986	319444	309651	300003	297948	301881
	Female	288962	313811	292380	—	302131	290176	314571	306506	299068	294945	295460
	Male	292938	296343	280128	—	309408	296735	321707	312512	307417	299784	298958
Left Cerebellum	All	17778	20923	18416	17998	17714	18727	19528	18199	19403	18476	18757
	Female	18977	21069	19241	—	19764	17192	21076	18423	18401	18395	17980
	Male	16602	18022	17168	—	17194	17248	20717	18105	18409	16536	17226
Brainstem	All	27096	29130	26956	26780	28287	25640	28828	27379	27960	25553	26491
	Female	27463	28848	27681	—	28300	26745	28845	28990	27381	27139	27025
	Male	27163	28464	26206	—	27798	24978	28388	27043	28780	24614	25868
Right Cerebrum	All	295001	308601	291450	303761	308314	293416	313900	315401	305698	300719	304358
	Female	291044	316755	296016	—	306840	293843	310840	300108	301616	297551	302233
	Male	301049	300094	279420	—	309071	292915	320032	308057	313880	303307	293519
Right Cerebellum	All	18144	19213	17636	17558	17612	17386	18657	17688	18100	17266	17653
	Female	18653	19311	18205	—	18868	16974	19524	18726	18522	17069	17353
	Male	17040	17775	16321	—	17340	17947	19426	17224	17727	17510	16860
	Male	2409	2407	2372	—	2347	2401	2634	2537	2391	2565	2430
WM hypointensity	All	4234	2812	4506	5279	3421	8155	2844	4517	3089	6484	6560
	Female	3527	2905	3926	—	3260	7418	3064	3437	3117	4991	5130
	Male	5133	3156	6093	—	3639	7257	2741	4894	3191	7709	8005
Optic Chiasm	All	319	334	332	315	345	313	314	324	284	327	307
	Female	367	337	306	—	350	329	270	340	315	327	358
	Male	302	300	343	—	290	367	296	345	320	367	347
Corpus Callosum Posterior	All	1381	1479	1373	1367	1460	1502	1445	1397	1448	1342	1431
	Female	1131	1479	1459	—	1457	1554	1459	1538	1409	1441	1168
	Male	1416	1461	1290	—	1468	1475	1451	1377	1438	1324	1379
Corpus Callosum Mid Posterior	All	735	902	761	710	840	625	871	765	837	580	711
	Female	751	934	827	—	882	614	946	970	857	651	762
	Male	741	753	700	—	829	689	854	743	797	520	671
Corpus Callosum Central	All	592	643	536	566	634	582	642	610	625	547	582
	Female	599	652	592	—	633	584	665	654	628	601	617
	Male	596	593	508	—	609	571	629	592	625	505	546
Corpus Callosum Mid Anterior	All	555	612	540	528	601	543	646	584	623	550	561
	Female	550	657	569	—	607	554	660	644	615	576	599
	Male	560	574	492	—	574	534	640	562	620	519	527
Corpus Callosum Anterior	All	1118	1236	1101	1117	1183	1164	1237	1154	1187	1111	1148
	Female	1080	1234	1167	—	1183	1167	1240	1206	1184	1226	1166
	Male	1150	1158	1006	—	1206	1166	1256	1159	1158	1021	1099

**Table 5:** Volumetric CSF information (in mm^3^) for each template based on FreeSurfer segmentations.

Region	Template	AD	CIE	FTD	LBD	MCI	Mixed	PD-CIE	PD-CI	SCI	V-AD	V-MCI
Left Lateral Ventricle	All	30239	16223	28500	29065	21887	37810	17055	23597	19002	31970	29117
	Female	28317	15402	24960	—	19157	37530	16573	18297	18365	26975	24155
	Male	31737	20534	34239	—	23506	38612	17183	24517	20908	35823	33251
Left Inf Lateral Ventricle	All	2112	590	1598	1477	986	2496	617	1278	735	2003	1475
	Female	1448	550	1224	—	776	1896	565	902	727	1300	1129
	Male	2082	739	1659	—	1063	2480	761	1270	953	2154	1670
3^rd^ Ventricle	All	2741	1743	2719	2658	2269	2987	1753	2622	1886	2910	2607
	Female	2378	1693	2409	—	1992	2816	1726	1764	1832	2375	2254
	Male	3027	2033	3056	—	2469	3168	1761	2753	2146	3432	3053
4^th^ Ventricle	All	2620	2351	2711	2403	2415	2717	1982	2371	2408	2589	2603
	Female	2352	2329	2587	—	2438	2754	1955	2052	2319	2663	2464
	Male	2573	2516	2724	—	2410	2603	2079	2457	2590	2653	2604
CSF	All	2224	1760	2149	2058	1903	2298	1762	2078	1894	2230	2018
	Female	2207	1645	2126	—	1824	2303	1792	1782	1782	1947	1925
	Male	2237	1733	2344	—	1969	2185	1820	2068	2068	2265	2016
Right Lateral Ventricle	All	28475	15074	25550	25397	19924	32426	16228	21808	17567	29175	25775
	Female	25694	14097	22697	—	17824	32040	15922	17923	16740	23267	23041
	Male	30456	19501	29515	—	21773	34284	16130	22301	20195	33219	29016
Right Inf Lateral Ventricle	All	1723	516	1490	1219	814	2090	590	1055	607	1950	1375
	Female	1364	456	1233	—	748	1682	462	723	607	1229	1045
	Male	1894	704	1652	—	905	2192	639	1173	844	2274	1570

**Fig. 9:**
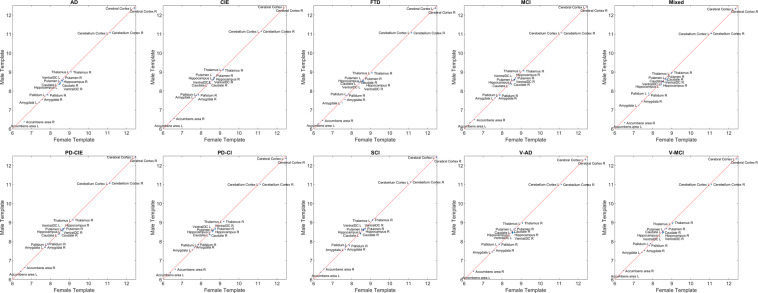
FreeSurfer based GM volumes for male and female templates for each diagnostic group. L: Left. R: Right.

Figure 9 compares GM volumes (log transformed) of male versus female templates. Note that since all templates have been linearly registered to the MNI-ICBM2009c template prior to the template creation step, all volumetric values reflect variabilities after accounting for intracranial volume differences and are not caused by potential head size differences between males and females. Data points below the reference line (shown in red) indicate lower values for the male template in comparison with the female template. In the AD and mixed templates, the nucleus accumbens areas bilaterally had lower volumes in the male templates. In the PD-CI template, most regions had slightly lower GM volumes in the male template.

As expected, mixed dementia, vascular MCI, and vascular AD templates had higher WM hypointensity volumes (corresponding to the WMHs on FLAIR and T2w sequences) on T1w templates (Table 4). Male templates for AD, FTD, PD-CI, V-MCI, and V-AD also had greater WM hypointensity volumes than the female templates (Table 4). The mixed template had the largest ventricles (Table 5), followed by V-AD and AD templates. As expected, CIE template had the smallest ventricles, followed by PD-CIE, and SCI. In all diagnostic groups, lateral ventricles were larger for the male templates in comparison with the female templates. This difference was most prominent in the V-AD group, for which the left and right lateral ventricles were 33% and 43% larger respectively for the male template (Table 5). Regarding asymmetry, in the FTD, V-MCI, and mixed templates, the left lateral ventricle was 12%, 13%, and 17% larger than the right lateral ventricle. This difference was more prominent in the male templates for FTD and V-MCI groups, whereas for the mixed group, the female template had greater asymmetry in the ventricles. All of these differences highlight the need for group-specific templates in multi-individual, multi-centric studies.

### Using Disease Appropriate Templates to Improve Registration

Use of age and disease appropriate templates can reduce both linear and nonlinear registration errors. We have previously shown that older subjects, those with larger ventricles, and high levels of WMHs have higher levels of linear registration failure rates when using young adult brain templates as the registration target for most widely used registration tools such as FSL, SPM, ANTs, Elastix, and MINC^5^. Using disease appropriate templates could be the solution to improve both linear and nonlinear registration for aged and diseased populations. Note that since all templates are in the same space (i.e. share a similar alignment to a pseudo-Talairach coordinate system), linear registration to one would be equivalent to linear registration to other templates without additional manipulation. As for nonlinear registration, these templates can be used as intermediate registration targets even in cases where the intended final application is to register all subjects to one healthy or younger average brain. Intermediate templates have been previously used for various registration tasks, particularly when there exists a large difference between source and target templates^35–38^. Disease appropriate average templates can be used as intermediate registration targets to improve nonlinear registration, using the following steps:

1. Linearly register patient brain image(s) to the disease appropriate template.
2. Nonlinearly register patient brain image(s) to the disease appropriate template.
3. Concatenate the nonlinear transformation with the precomputed nonlinear transformation between the two average templates.
4. If necessary, the registration can be refined by performing another nonlinear registration between the nonlinearly transformed image and the average template. Concatenate this additional transformation with the previous two.

**Fig. 10:**
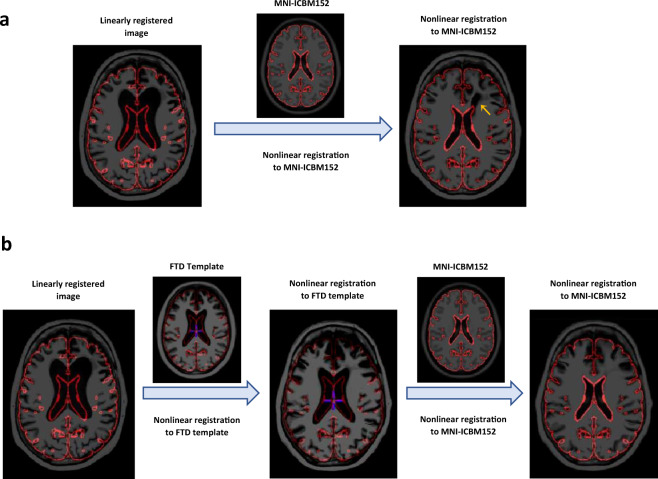
An example of T1-weighted scan of an individual with frontotemporal dementia (FTD) that was nonlinearly registered to MNI-ICBM152 average template directly (**a**) and using a disease appropriate template as an intermediate registration target (**b**). The red contour shows the outline of MNI-ICBM152 template, and can be used to assess registration accuracy. The orange arrow shows the areas of gross registration failure.

Figure 10 demonstrates how using a disease appropriate average template can improve nonlinear registration. Panel a shows a nonlinear registration scenario in which the brain of an individual with FTD has been nonlinearly registered directly to the MNI-ICBM152 average template using ANTs diffeomorphic registration tool^39^. The red contours consistently show the outline of the MNI-ICBM152 brain and can be used to assess the quality of the nonlinear registration. In a perfectly registered image, the contours of MNI-ICBM152 should match the contours of the nonlinearly deformed image (shown in the last columns on the right). The orange arrow shows the areas of gross registration failure, where ANTs has not been able to accurately register the ventricles of the subject to MNI-ICBM152. This is a common occurrence in dementia patients with large ventricles and gross atrophy. Panel b shows registration results for the same individual, which was first nonlinearly registered to a disease appropriate FTD template, and then nonlinearly registered to the MNI-ICBM152. Comparing the two deformed images (last columns on the right), we can see that when the FTD template was used as an intermediate registration target, ANTs was able to accurately register the ventricles.

## Supplementary information

Supplementary Materials

## Data Availability

The scripts for generating unbiased average templates are publicly available at https://github.com/vfonov/nist_mni_pipelines.

## References

[1] Ashburner, J. *et al*. SPM12 manual. *Wellcome Trust Cent. Neuroimaging Lond. UK* (2014).

[2] Aubert-Broche B (2013). A new method for structural volume analysis of longitudinal brain MRI data and its application in studying the growth trajectories of anatomical brain structures in childhood. NeuroImage.

[3] Jenkinson M, Beckmann CF, Behrens TE, Woolrich MW, Smith SM (2012). Fsl. Neuroimage.

[4] Mateos-Pérez JM (2018). Structural neuroimaging as clinical predictor: A review of machine learning applications. NeuroImage Clin..

[5] Dadar M, Fonov VS, Collins DL, Initiative ADN (2018). A comparison of publicly available linear MRI stereotaxic registration techniques. NeuroImage.

[6] Ridwan AR (2021). Development and evaluation of a high performance T1-weighted brain template for use in studies on older adults. Hum. Brain Mapp..

[7] Dadar M, Manera AL, Fonov VS, Ducharme S, Collins DL (2021). MNI-FTD templates, unbiased average templates of frontotemporal dementia variants. Sci. Data.

[8] Van Hecke W (2011). The effect of template selection on diffusion tensor voxel-based analysis results. NeuroImage.

[9] Avants B, Tustison N (2018). figshare.

[10] Klein A (2017). Harvard Dataverse..

[11] Kötter R (2001). A probabilistic atlas and reference system for the human brain: International Consortium for Brain Mapping (ICBM). Philos. Trans. R. Soc. Lond. B. Biol. Sci..

[12] Fonov V (2011). Unbiased average age-appropriate atlases for pediatric studies. NeuroImage.

[13] Yoon U, Fonov VS, Perusse D, Evans AC (2009). The effect of template choice on morphometric analysis of pediatric brain data. NeuroImage.

[14] Xiao Y (2015). Multi-contrast unbiased MRI atlas of a Parkinson’s disease population. Int. J. Comput. Assist. Radiol. Surg..

[15] Guo X-Y (2021). Development and evaluation of a T1 standard brain template for Alzheimer disease. Quant. Imaging Med. Surg..

[16] Chertkow H (2019). The Comprehensive Assessment of Neurodegeneration and Dementia: Canadian Cohort Study. Can. J. Neurol. Sci..

[17] Duchesne S (2019). Structural and functional multi-platform MRI series of a single human volunteer over more than fifteen years. Sci. Data.

[18] Altmann A, Tian L, Henderson VW, Greicius MD (2014). & Investigators, A. D. N. I. Sex modifies the APOE-related risk of developing Alzheimer disease. Ann. Neurol..

[19] Tierney MC, Curtis AF, Chertkow H, Rylett RJ (2017). Integrating sex and gender into neurodegeneration research: A six-component strategy. Alzheimers Dement. Transl. Res. Clin. Interv..

[20] Bellou V, Belbasis L, Tzoulaki I, Evangelou E, Ioannidis JP (2016). Environmental risk factors and Parkinson’s disease: an umbrella review of meta-analyses. Parkinsonism Relat. Disord..

[21] Pieruccini‐Faria, F. *et al*. Gait variability across neurodegenerative and cognitive disorders: Results from the Canadian Consortium of Neurodegeneration in Aging (CCNA) and the Gait and Brain Study. *Alzheimers Dement*. **n/a** (2021). 10.1002/alz.12298 PMC8451764 33590967

[22] Dadar, M. *et al*. *White Matter Hyperintensity Distribution Differences in Aging and Neurodegenerative Disease Cohorts*. 2021.11.23.469690 10.1101/2021.11.23.469690 (2021). 10.1016/j.nicl.2022.103204 PMC9668605 36155321

[23] Duchesne S (2019). The Canadian Dementia Imaging Protocol: Harmonizing National Cohorts. J. Magn. Reson. Imaging.

[24] Coupe P (2008). An Optimized Blockwise Nonlocal Means Denoising Filter for 3-D Magnetic Resonance Images. IEEE Trans. Med. Imaging.

[25] Sled JG, Zijdenbos AP, Evans AC (1998). A nonparametric method for automatic correction of intensity nonuniformity in MRI data. IEEE Trans. Med. Imaging.

[26] Manera AL, Dadar M, Fonov V, Collins DL (2020). CerebrA, registration and manual label correction of Mindboggle-101 atlas for MNI-ICBM152 template. Sci. Data.

[27] Fonov V, Evans A, McKinstry R, Almli C, Collins D (2009). Unbiased nonlinear average age-appropriate brain templates from birth to adulthood. NeuroImage.

[28] Dadar M, Manera AL, Fonov VS, Ducharme S, Collins DL (2021). MNI-FTD Templates: Unbiased Average Templates of Frontotemporal Dementia Variants. Sci. Data.

[29] Collins DL, Evans AC (1997). Animal: validation and applications of nonlinear registration-based segmentation. Int. J. Pattern Recognit. Artif. Intell..

[30] Fischl B (2012). FreeSurfer. Neuroimage.

[31] Neelin P, MacDonald D, Collins DL, Evans AC (1998). The MINC file format: from bytes to brains. NeuroImage.

[32] Vincent RD (2016). MINC 2.0: a flexible format for multi-modal images. Front. Neuroinformatics.

[33] Dadar M, Camicioli R, Duchesne S (2021). Multi-Sequence Average Templates for Aging and Neurodegenerative Disease Populations..

[34] Dadar M, Camicioli R, Duchesne S (2021). Multi-Sequence Average Templates for Aging and Neurodegenerative Disease Populations..

[35] Christensen, G. E. & He, J. Consistent nonlinear elastic image registration. in *Proceedings IEEE Workshop on Mathematical Methods in Biomedical Image Analysis (MMBIA 2001)* 37–43, 10.1109/MMBIA.2001.991697 (2001).

[36] Jia H, Yap P-T, Wu G, Wang Q, Shen D (2011). Intermediate templates guided groupwise registration of diffusion tensor images. NeuroImage.

[37] Xiao Y (2019). An accurate registration of the BigBrain dataset with the MNI PD25 and ICBM152 atlases. Sci. Data.

[38] Jia H (2012). Directed graph based image registration. Comput. Med. Imaging Graph..

[39] Avants BB, Tustison N, Song G (2009). Advanced normalization tools (ANTS). Insight J.

