# Supplementary material for: Multi sequence average templates for aging and neurodegenerative disease populations
Source: Sci Data. 2022 May 27;9:238. doi: 10.1038/s41597-022-01341-2 (PMC9142602; doi:10.1038/s41597-022-01341-2)
Supplement: Supplementary file 1 — Supplementary Materials [file 41597_2022_1341_MOESM1_ESM.pdf]

Table of Contents

Figure S.1. Axial slices of average AD templates …… 2

Figure S.2. Axial slices of average CIE templates …… 3

Figure S.3. Axial slices of average FTD templates …… 4

Figure S.4. Axial slices of average LBD templates …… 5

Figure S.5. Axial slices of average MCI templates …… 6

Figure S.6. Axial slices of average Mixed templates …… 7

Figure S.7. Axial slices of average PD-CI templates …… 8

Figure S.8. Axial slices of average PD-CIE templates …… 9

Figure S.9. Axial slices of average SCI templates …… 10

Figure S.10. Axial slices of average V-AD templates …… 11

Figure S.11. Axial slices of average V-MCI templates …… 12

Figure S.12. Axial slices of average Female AD templates …… 13

Figure S.13. Axial slices of average Male AD templates …… 14

Figure S.14. Axial slices of average Female CIE templates …… 15

Figure S.15. Axial slices of average Male CIE templates …… 16

Figure S.16. Axial slices of average Female FTD templates …… 17

Figure S.17. Axial slices of average Male FTD templates …… 18

Figure S.18. Axial slices of average Female MCI templates …… 19

Figure S.19. Axial slices of average Male MCI templates …… 20

Figure S.20. Axial slices of average Female Mixed templates …… 21

Figure S.21. Axial slices of average Male Mixed templates …… 22

Figure S.22. Axial slices of average Female PD-CI templates …… 23

Figure S.23. Axial slices of average Male PD-CI templates …… 24

Figure S.24. Axial slices of average Female PD-CIE templates …… 25

Figure S.25. Axial slices of average Male PD-CIE templates …… 26

Figure S.26. Axial slices of average Female SCI templates …… 27

Figure S.27. Axial slices of average Male SCI templates …… 28

Figure S.28. Axial slices of average Female V-AD templates …… 29

Figure S.29. Axial slices of average Male V-AD templates …… 30

Figure S.30. Axial slices of average Female V-MCI templates …… 31

Figure S.31. Axial slices of average Male V-MCI templates …… 32

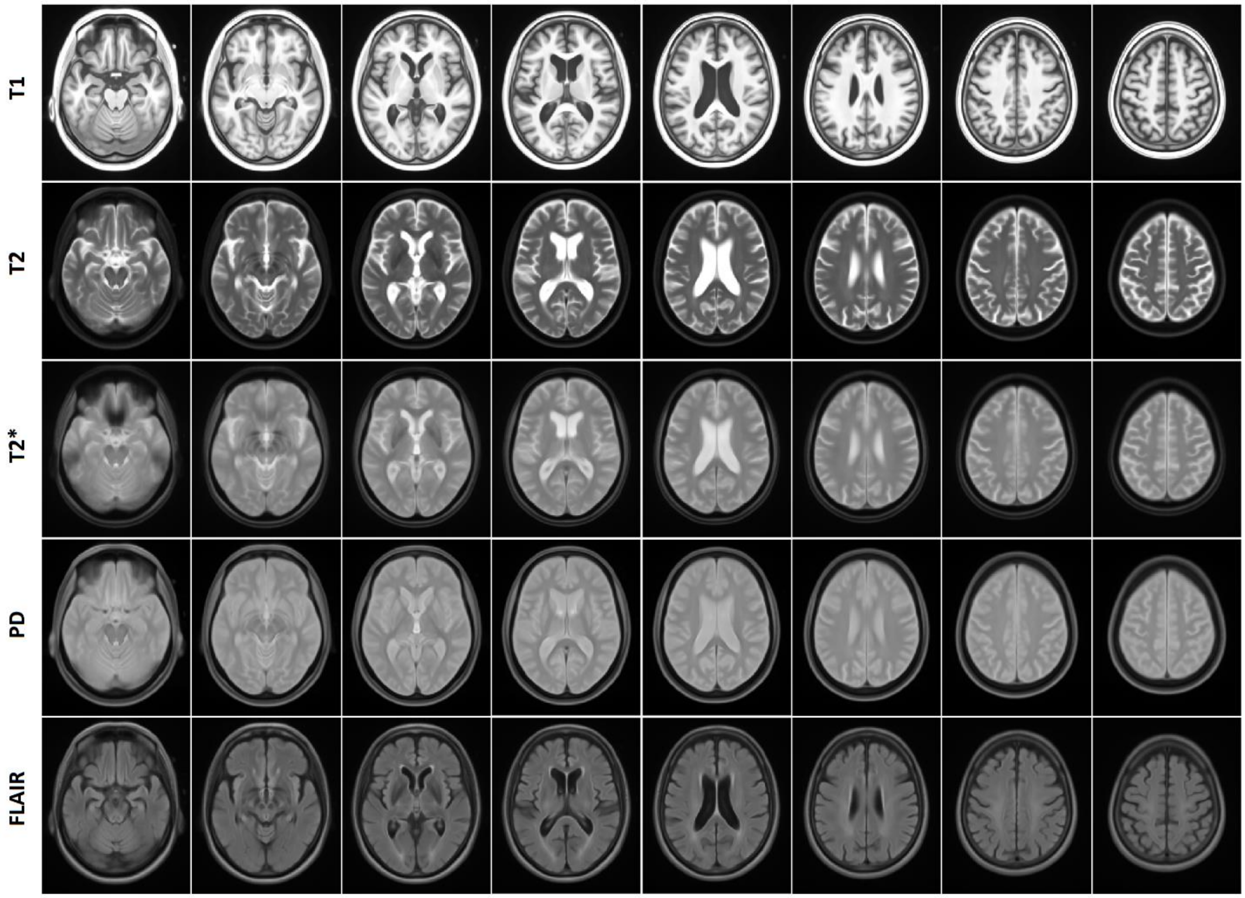

Figure S.1. Axial slices of average AD templates for T1, T2, T2*, PD, and FLAIR sequences.

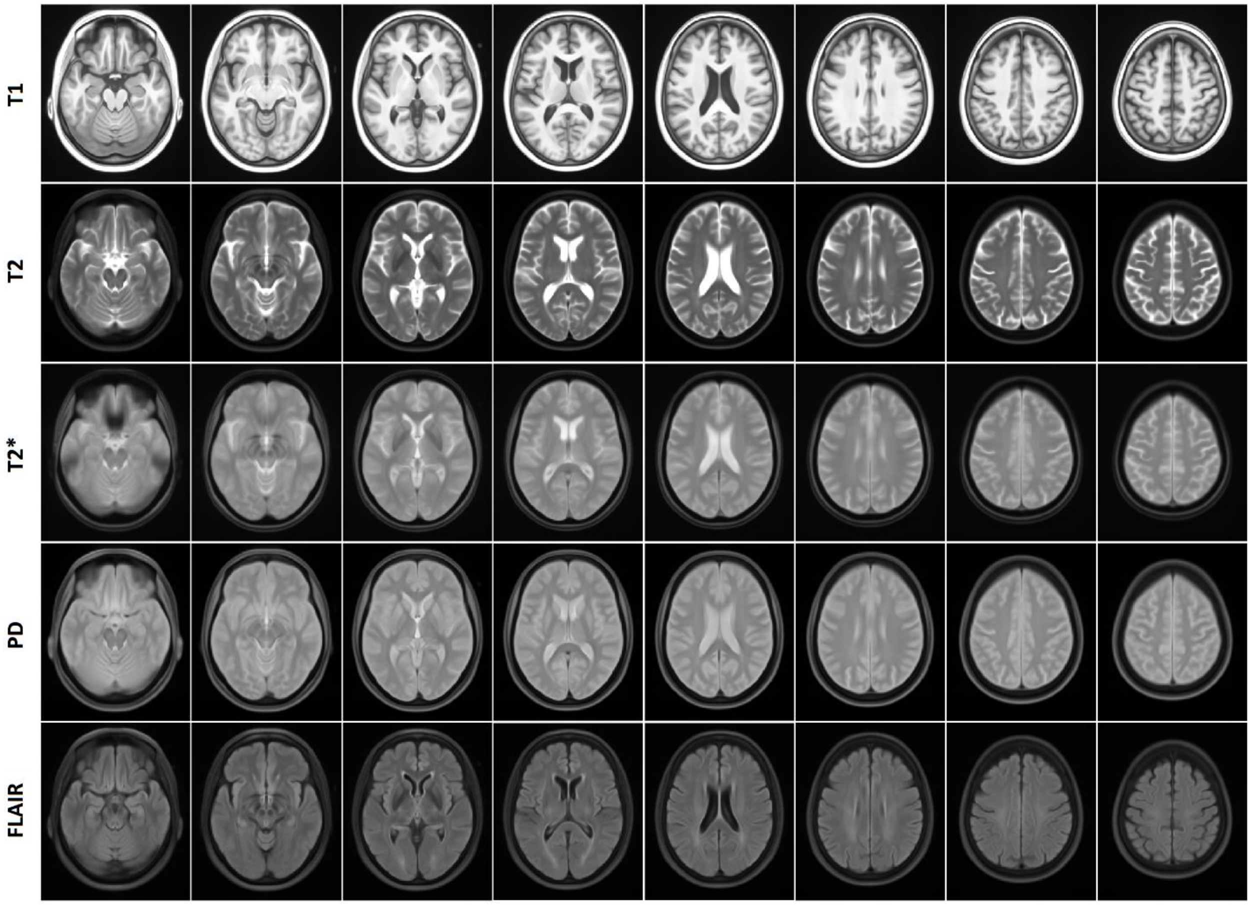

Figure S.2. Axial slices of average CIE templates for T1, T2, T2*, PD, and FLAIR sequences.

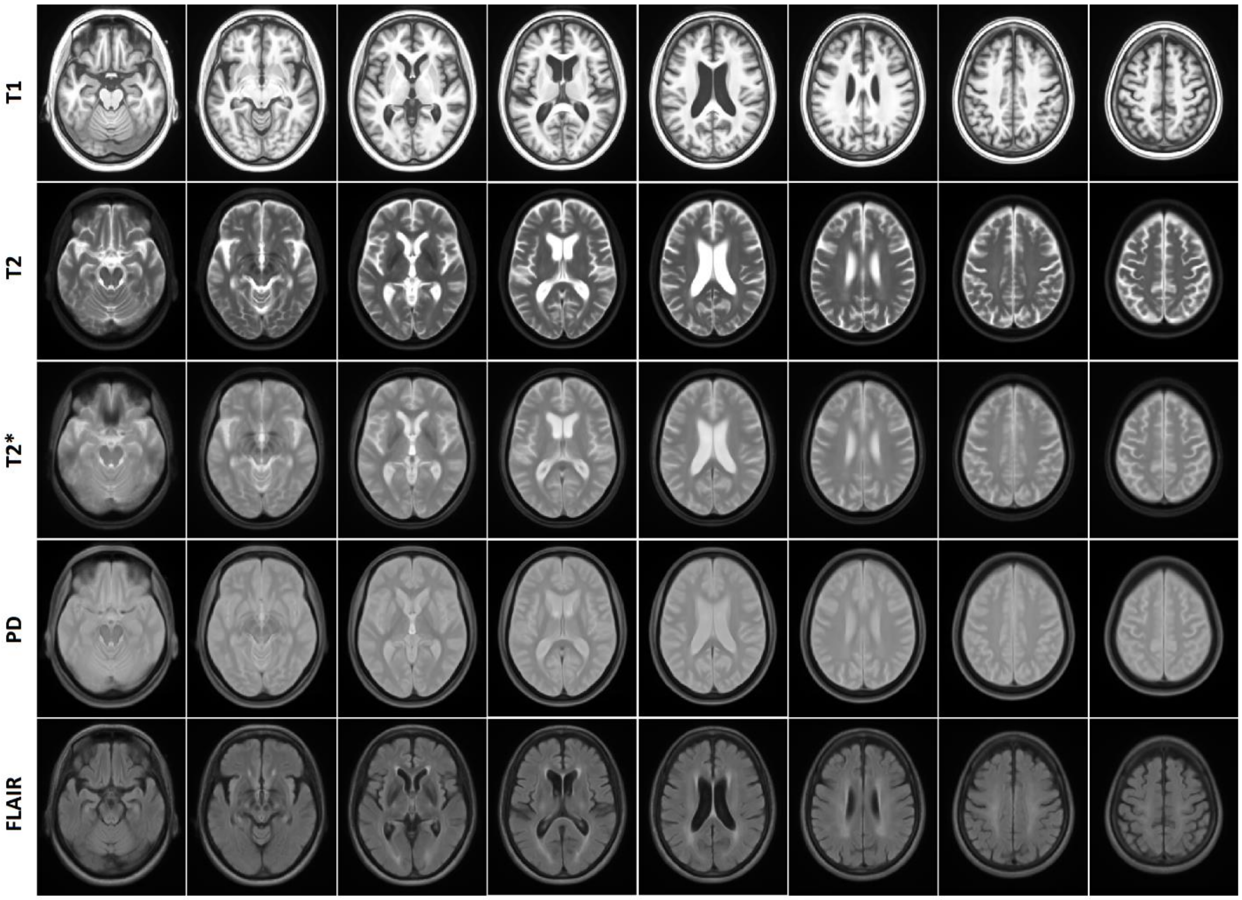

Figure S.3. Axial slices of average FTD templates for T1, T2, T2*, PD, and FLAIR sequences.

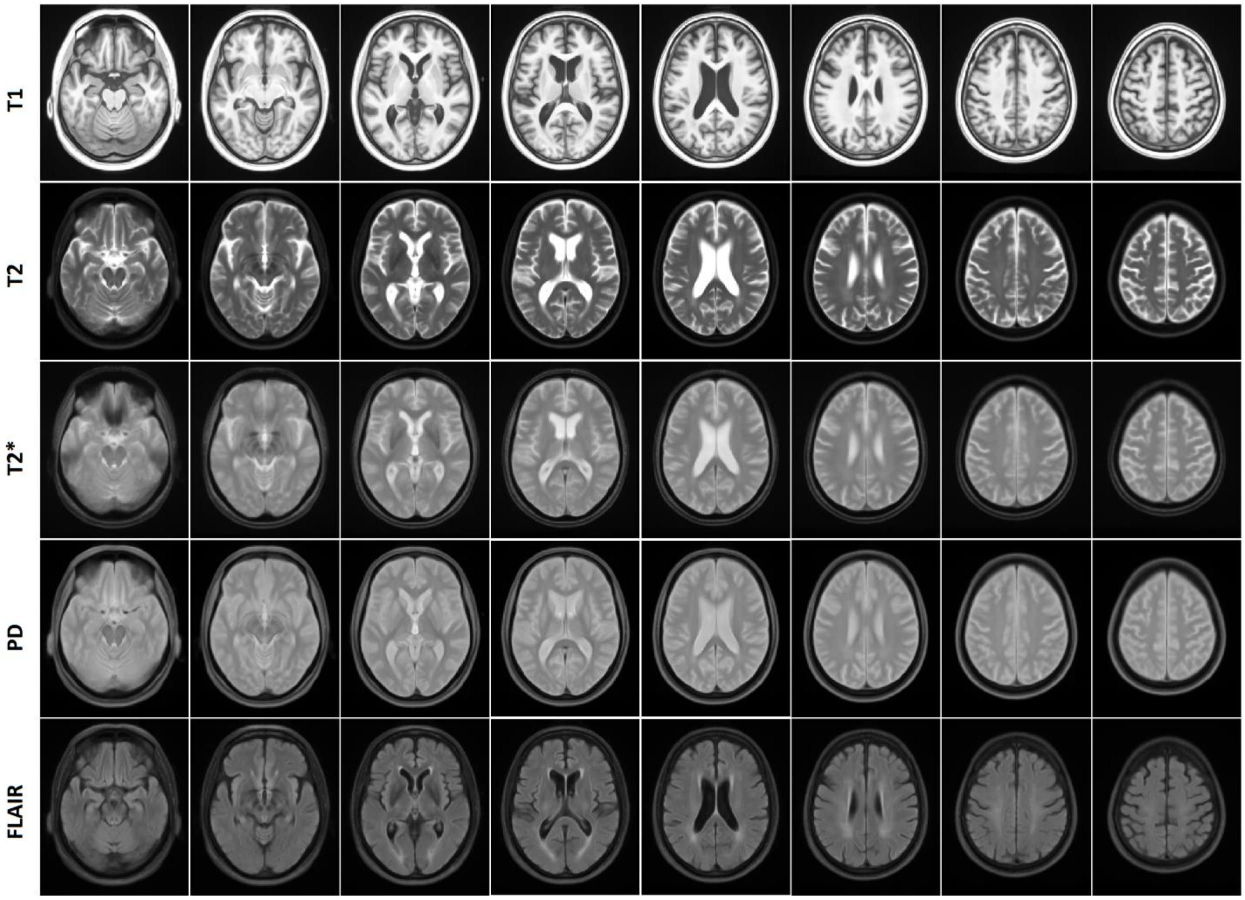

Figure S.4. Axial slices of average LBD templates for T1, T2, T2*, PD, and FLAIR sequences.

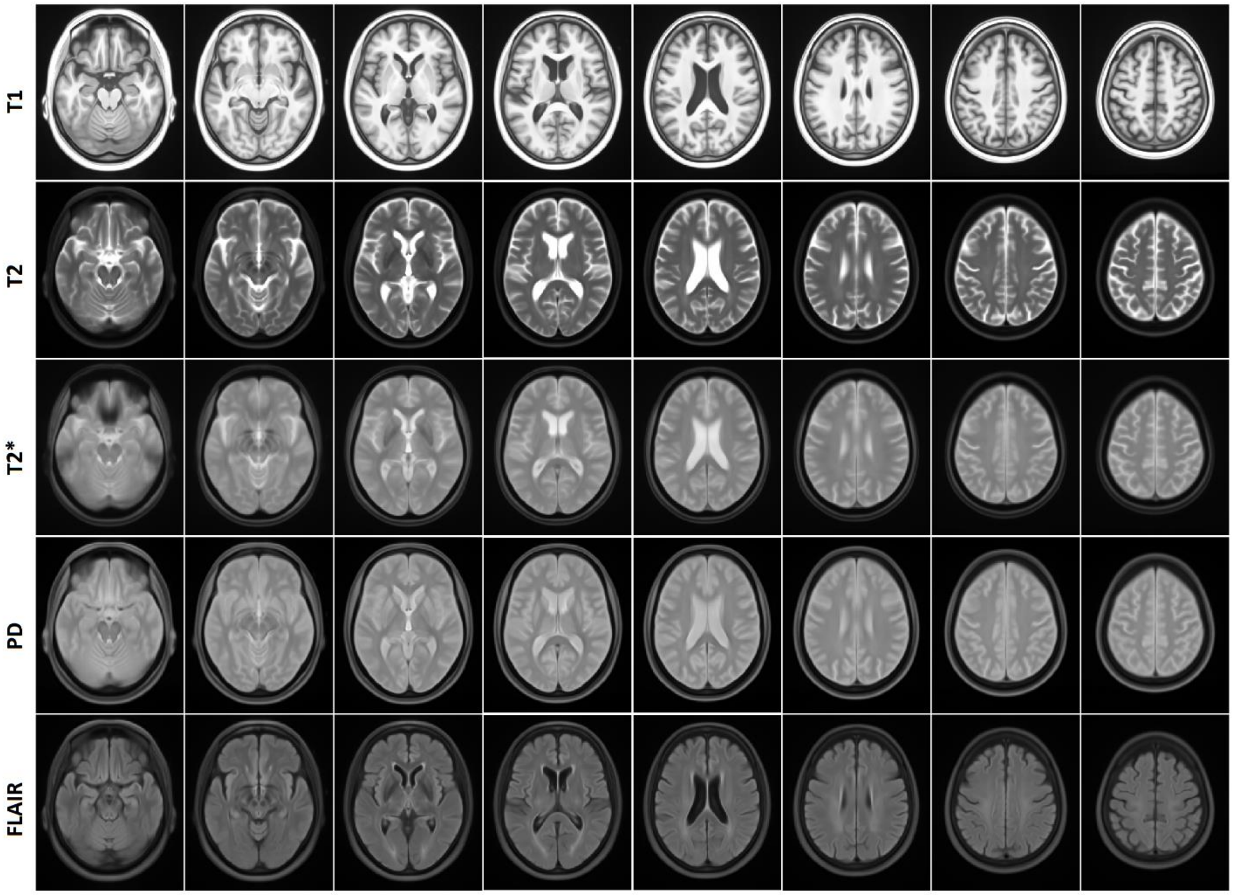

Figure S.5. Axial slices of average MCI templates for T1, T2, T2*, PD, and FLAIR sequences.

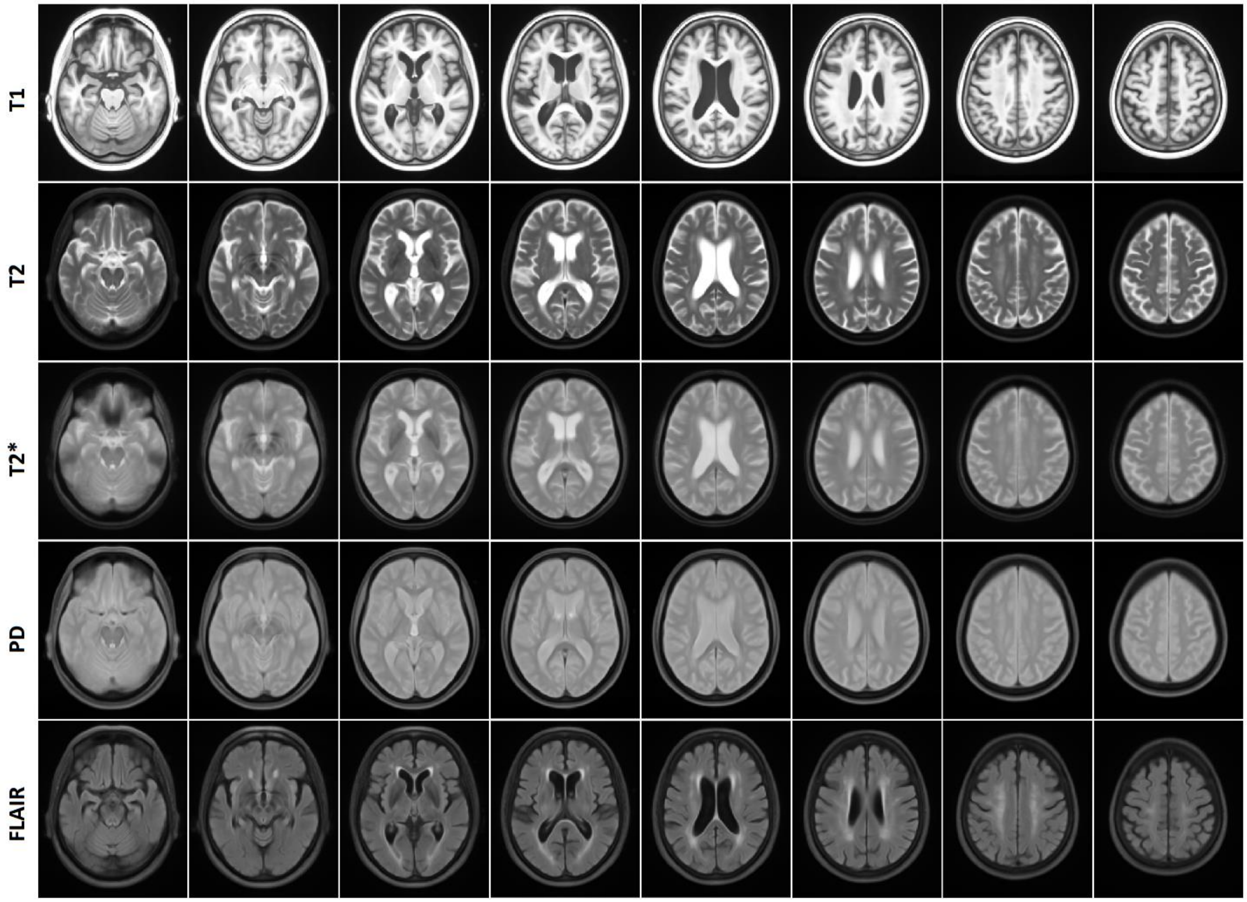

Figure S.6. Axial slices of average Mixed dementia templates for T1, T2, T2*, PD, and FLAIR sequences.

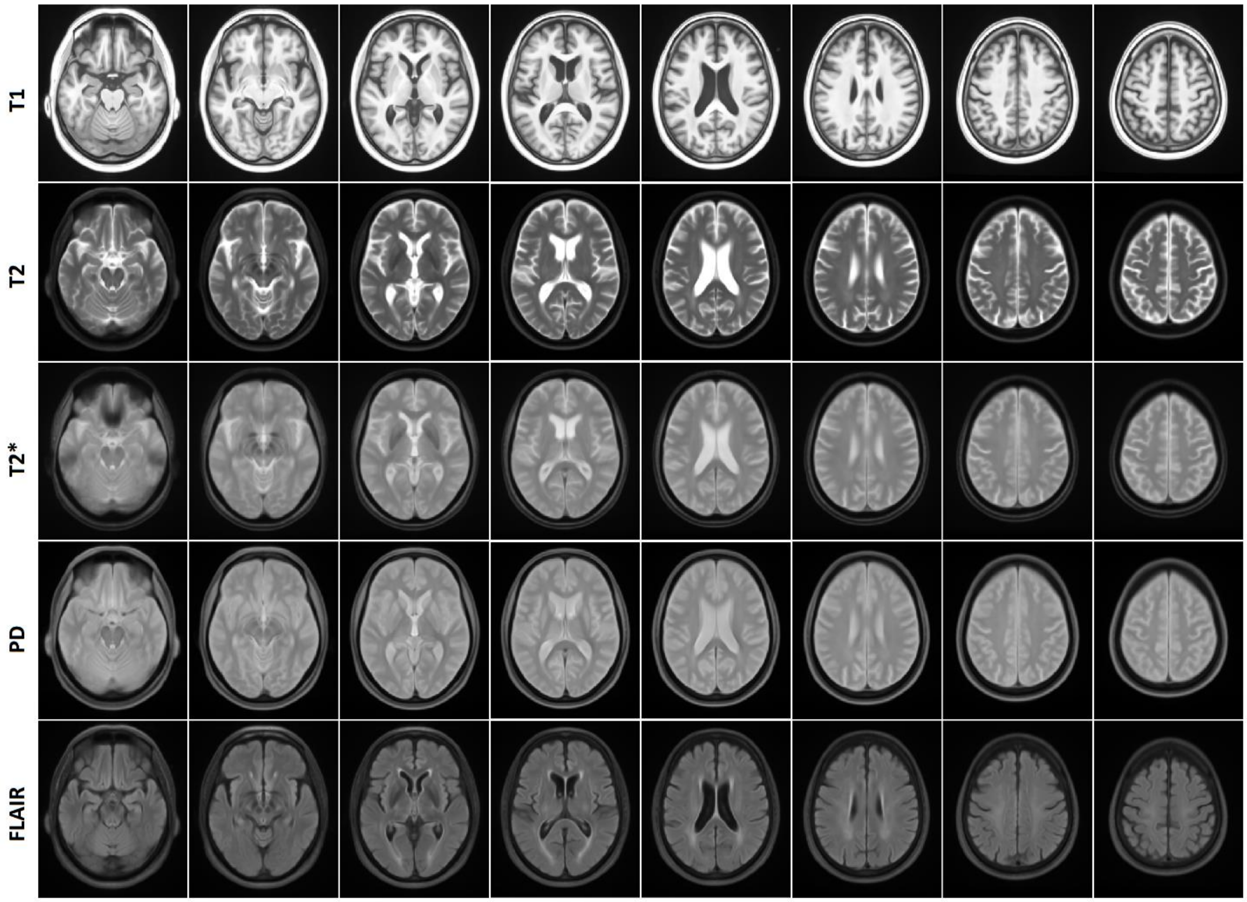

Figure S.7. Axial slices of average PD-CI templates for T1, T2, T2*, PD, and FLAIR sequences.

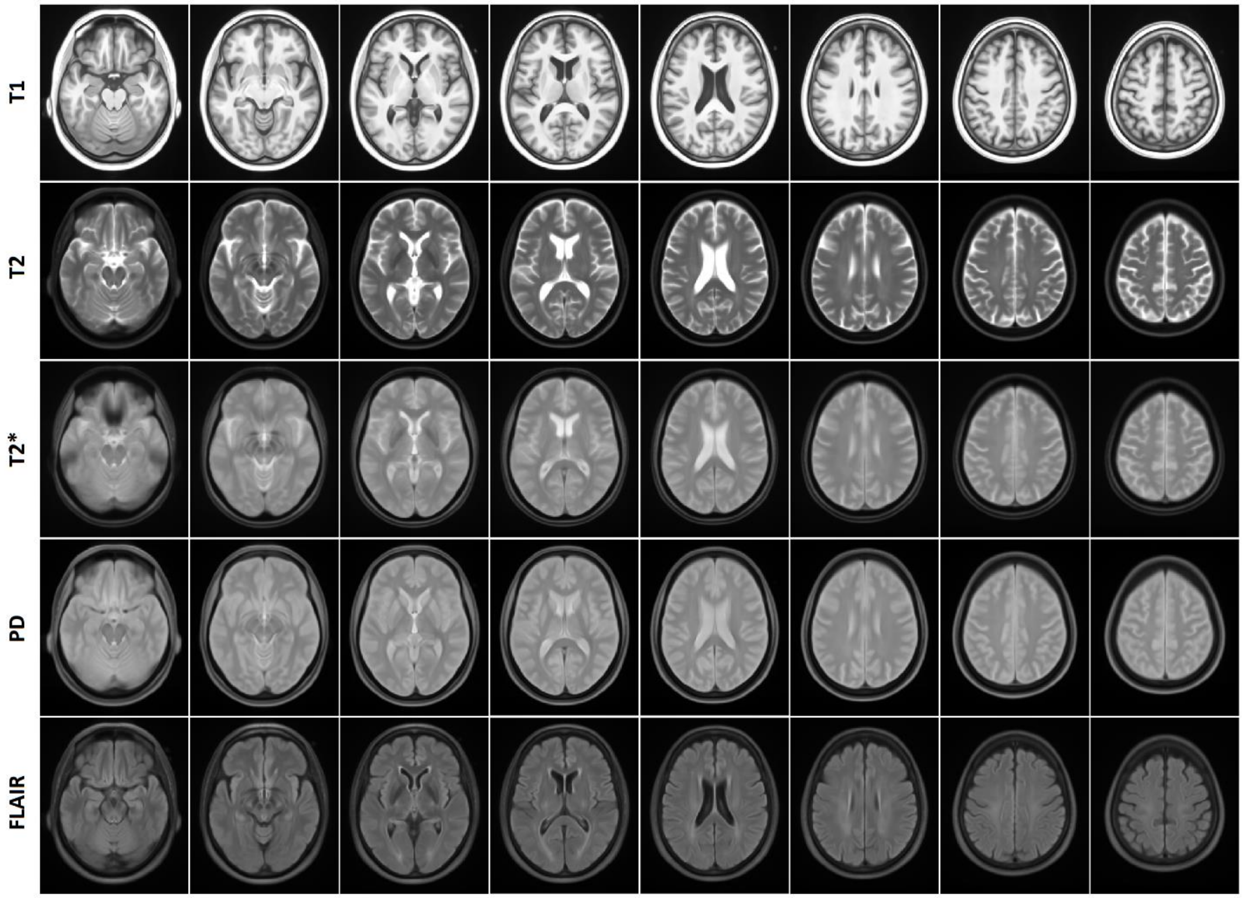

Figure S.8. Axial slices of average PD-CIE templates for T1, T2, T2*, PD, and FLAIR sequences.

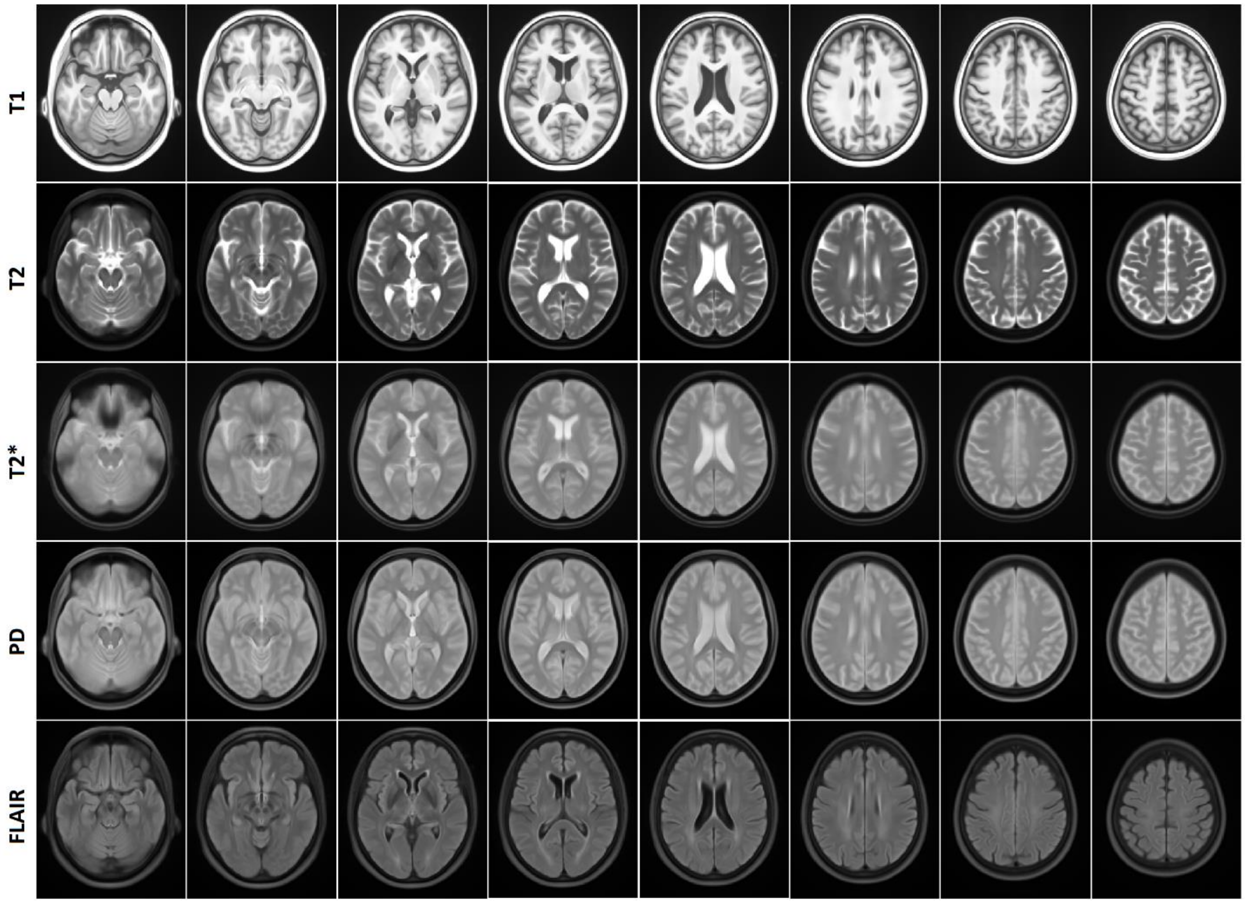

Figure S.9. Axial slices of average SCI templates for T1, T2, T2*, PD, and FLAIR sequences.

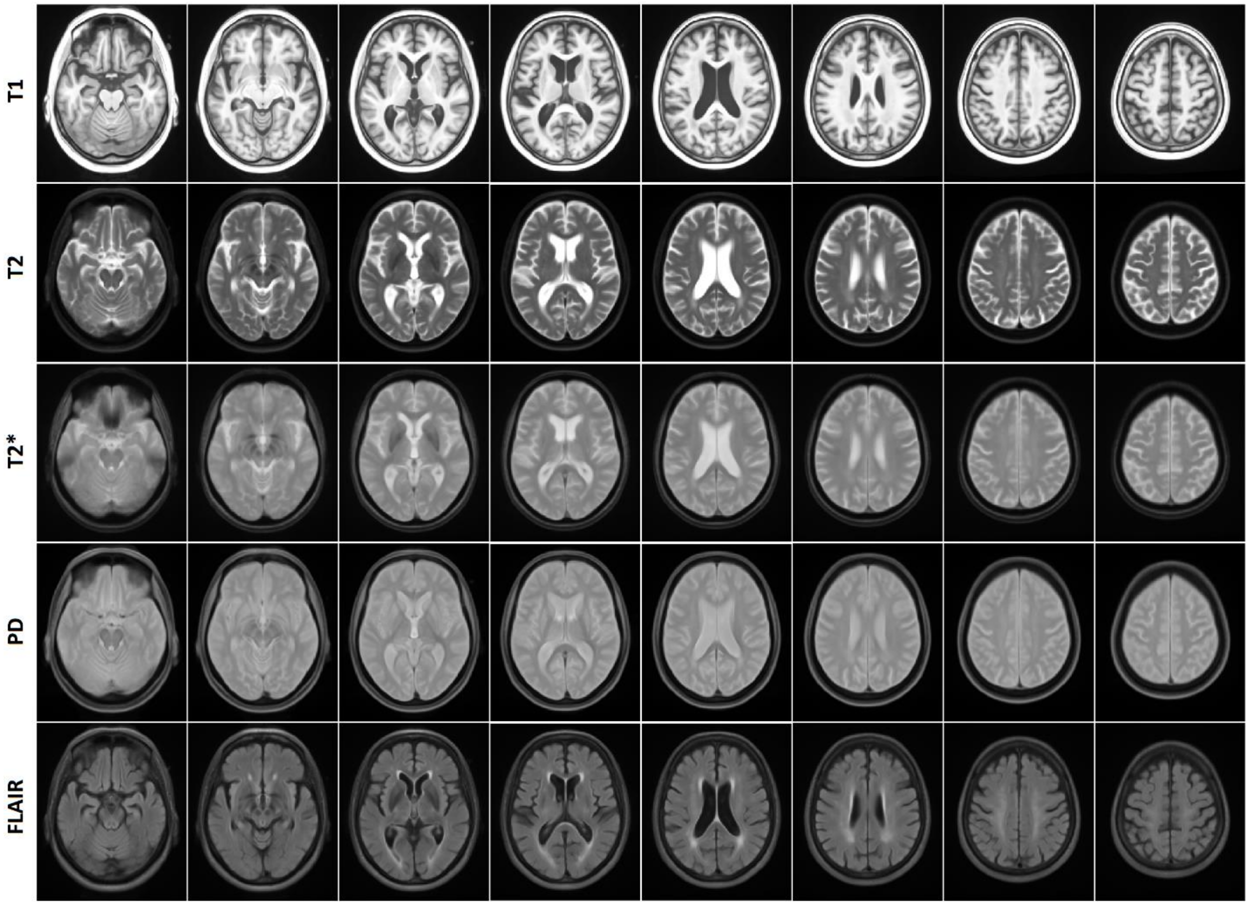

Figure S.10. Axial slices of average V-AD templates for T1, T2, T2*, PD, and FLAIR sequences.

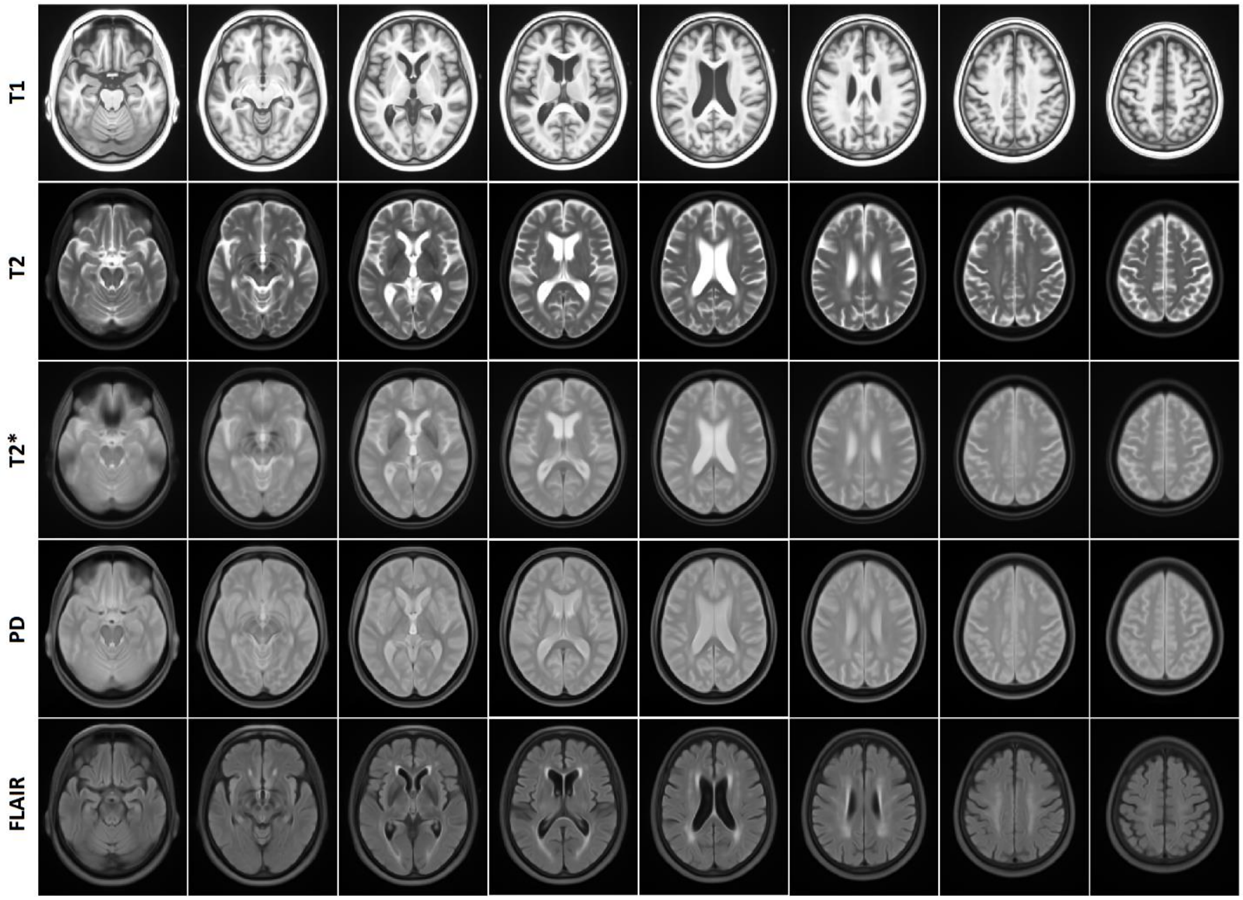

Figure S.11. Axial slices of average V-MCI templates for T1, T2, T2*, PD, and FLAIR sequences.

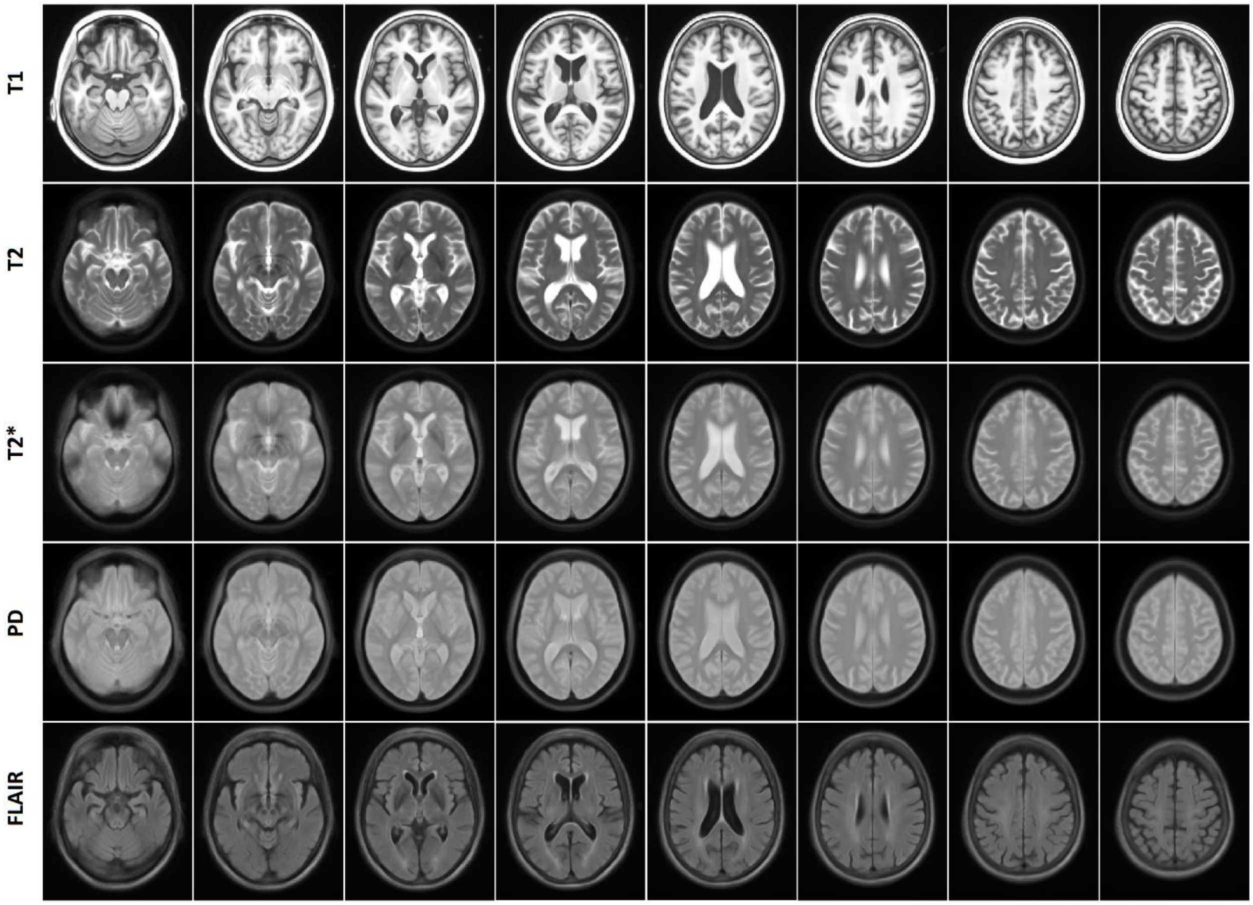

Figure S.12. Axial slices of average Female AD templates for T1, T2, T2*, PD, and FLAIR sequences.

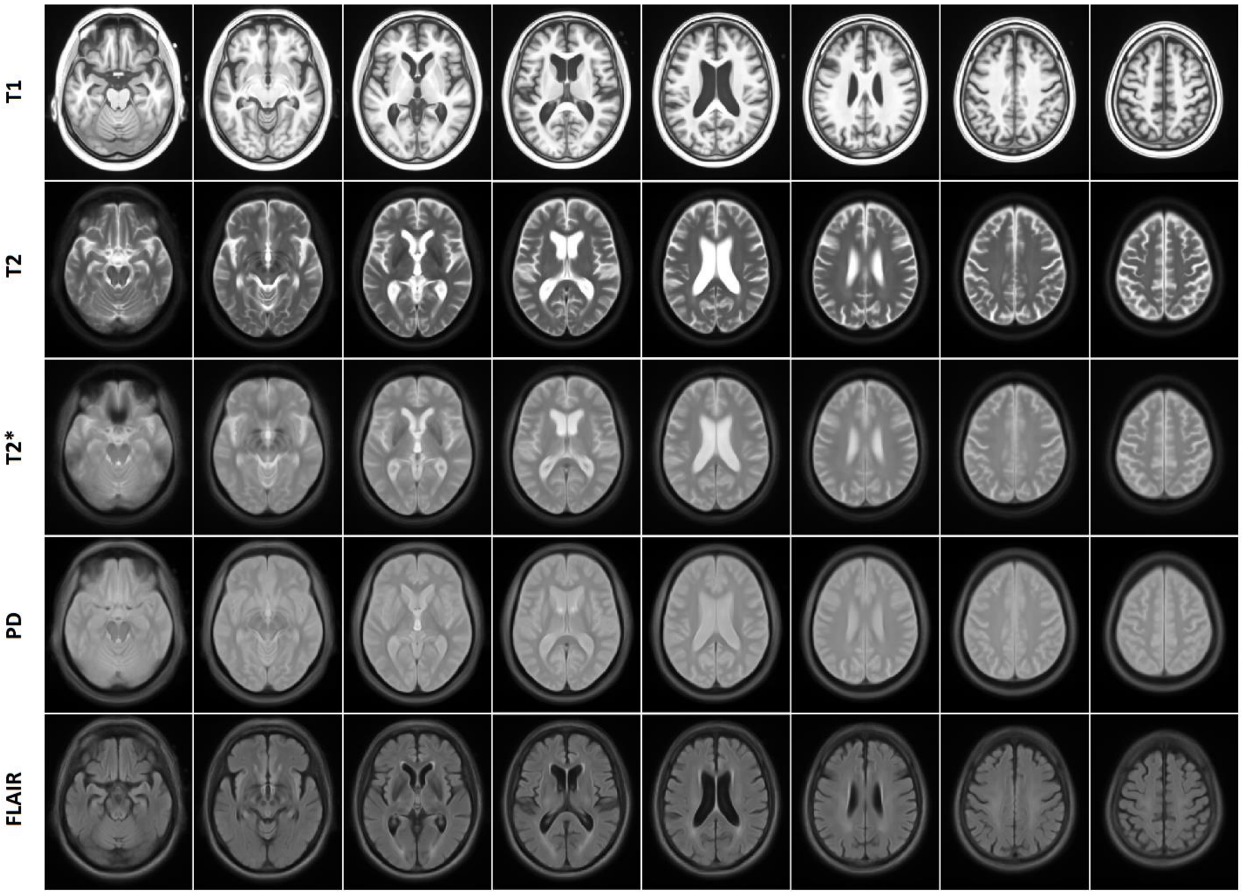

Figure S.13. Axial slices of average Male AD templates for T1, T2, T2*, PD, and FLAIR sequences.

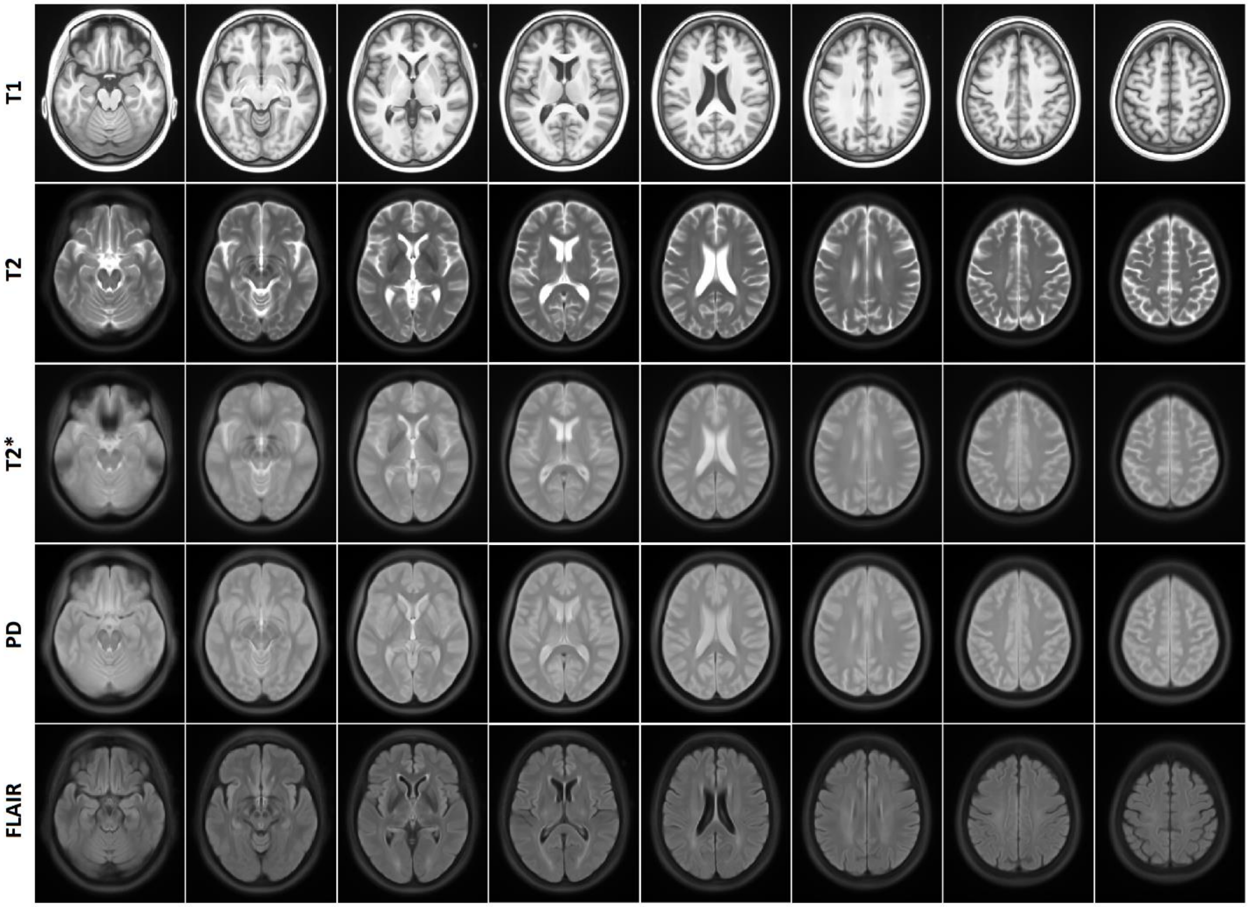

Figure S.14. Axial slices of average Female CIE templates for T1, T2, T2*, PD, and FLAIR sequences.

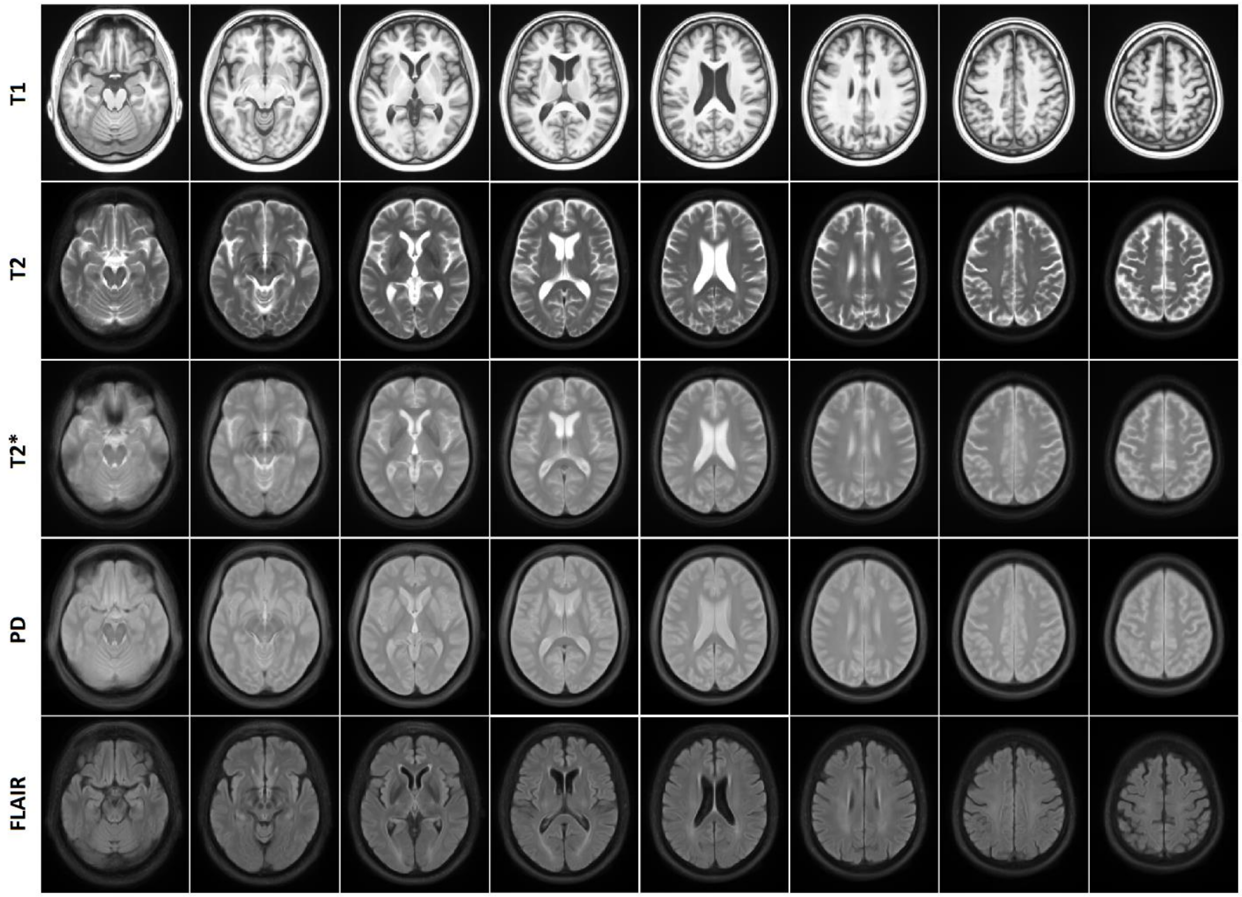

Figure S.15. Axial slices of average Male CIE templates for T1, T2, T2*, PD, and FLAIR sequences.

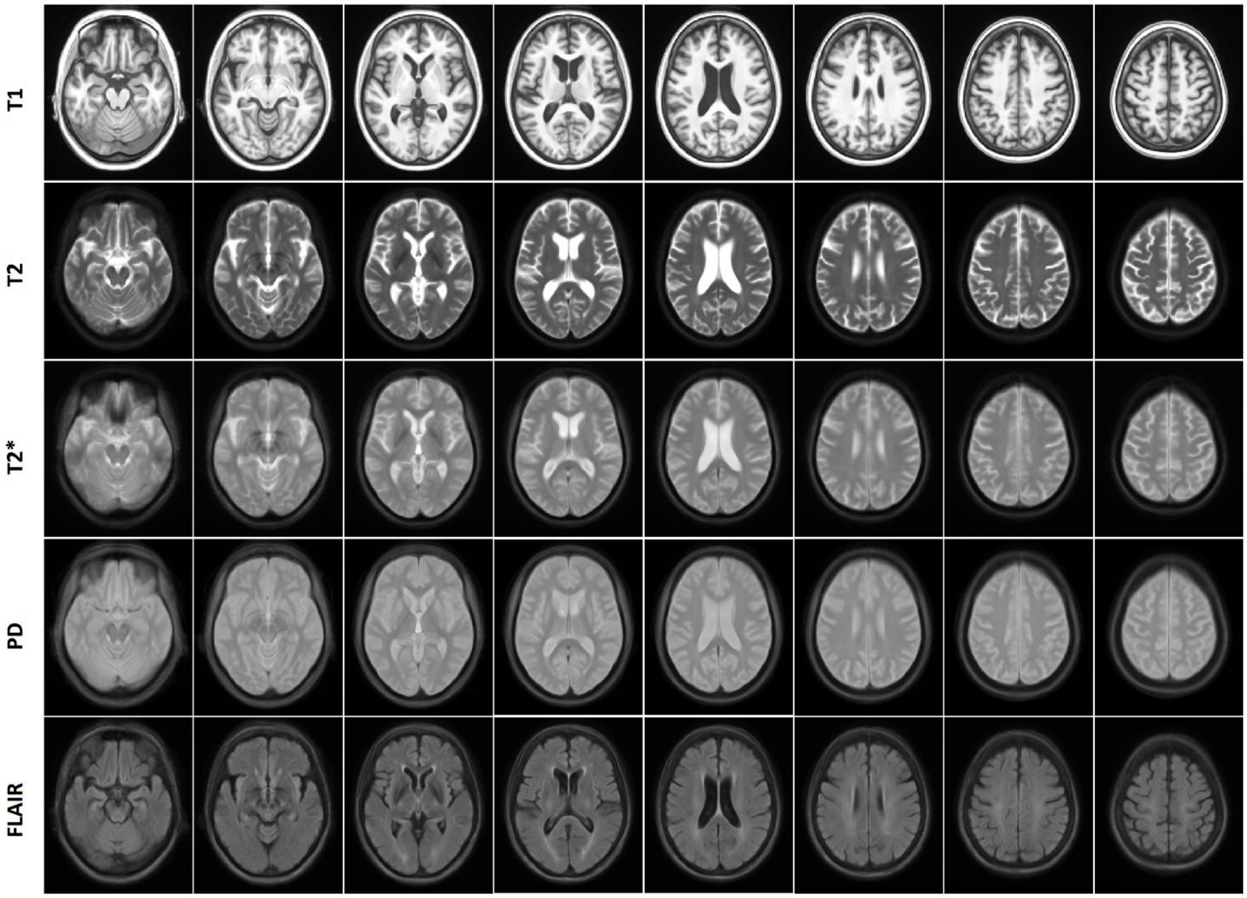

Figure S.16. Axial slices of average Female FTD templates for T1, T2, T2*, PD, and FLAIR sequences.

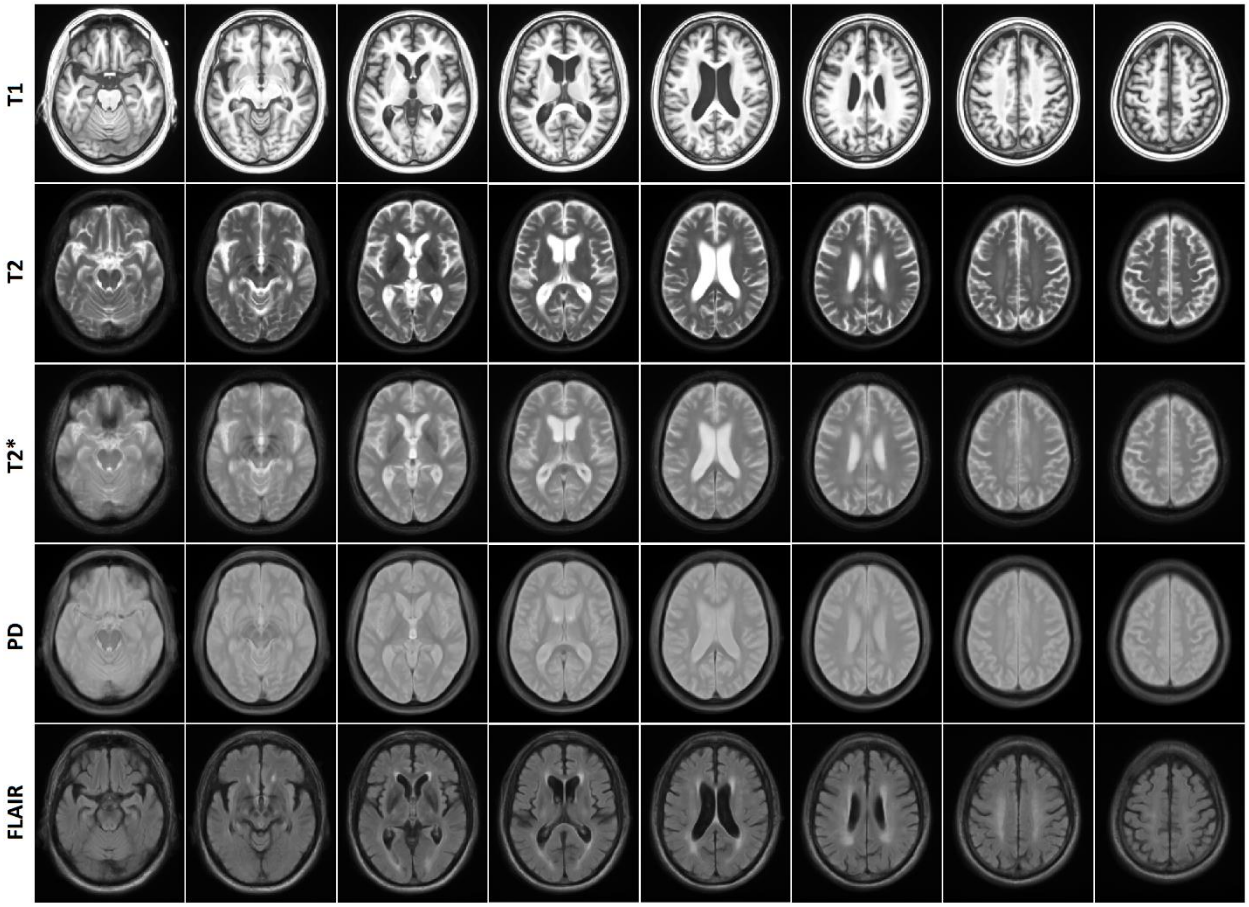

Figure S.17. Axial slices of average Male FTD templates for T1, T2, T2*, PD, and FLAIR sequences.

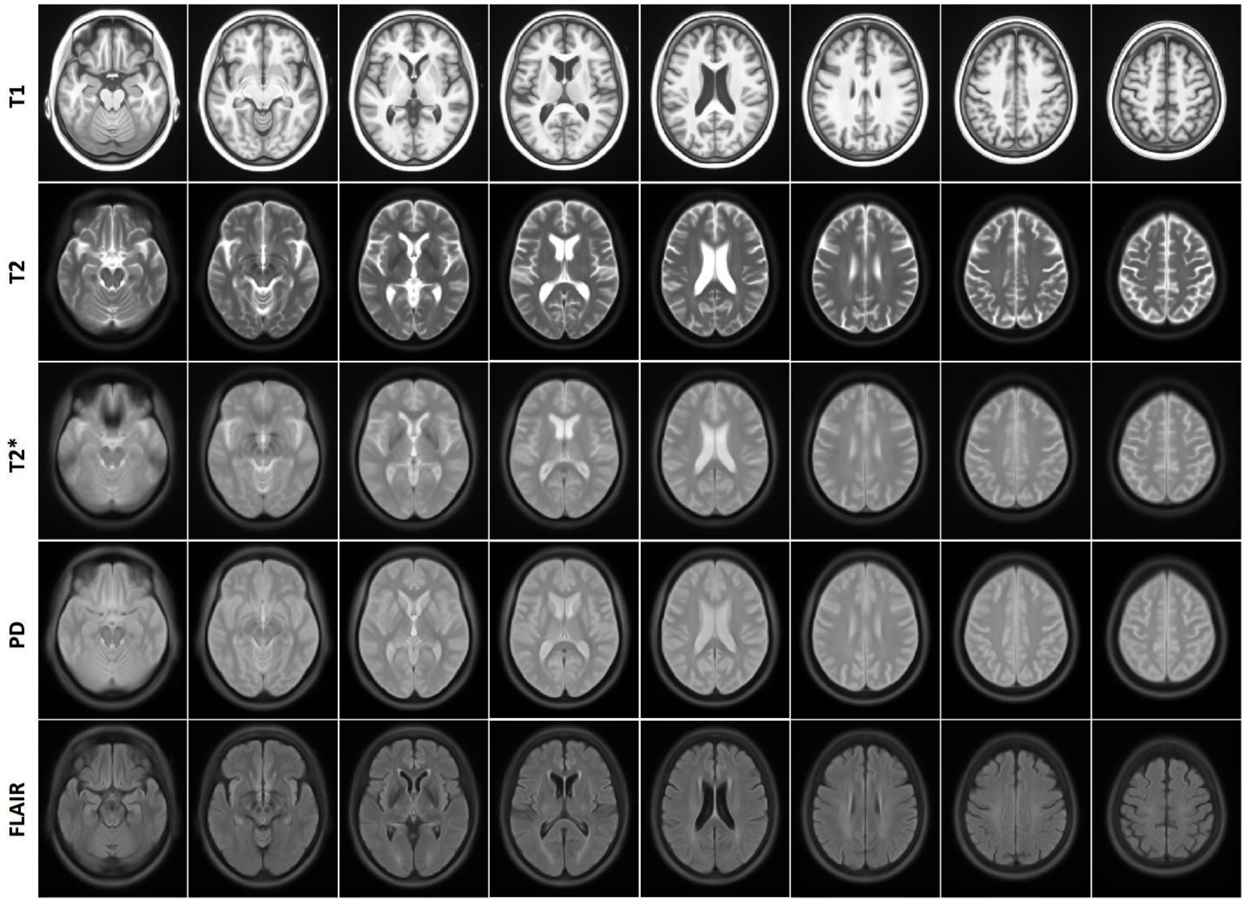

Figure S.18. Axial slices of average Female MCI templates for T1, T2, T2*, PD, and FLAIR sequences.

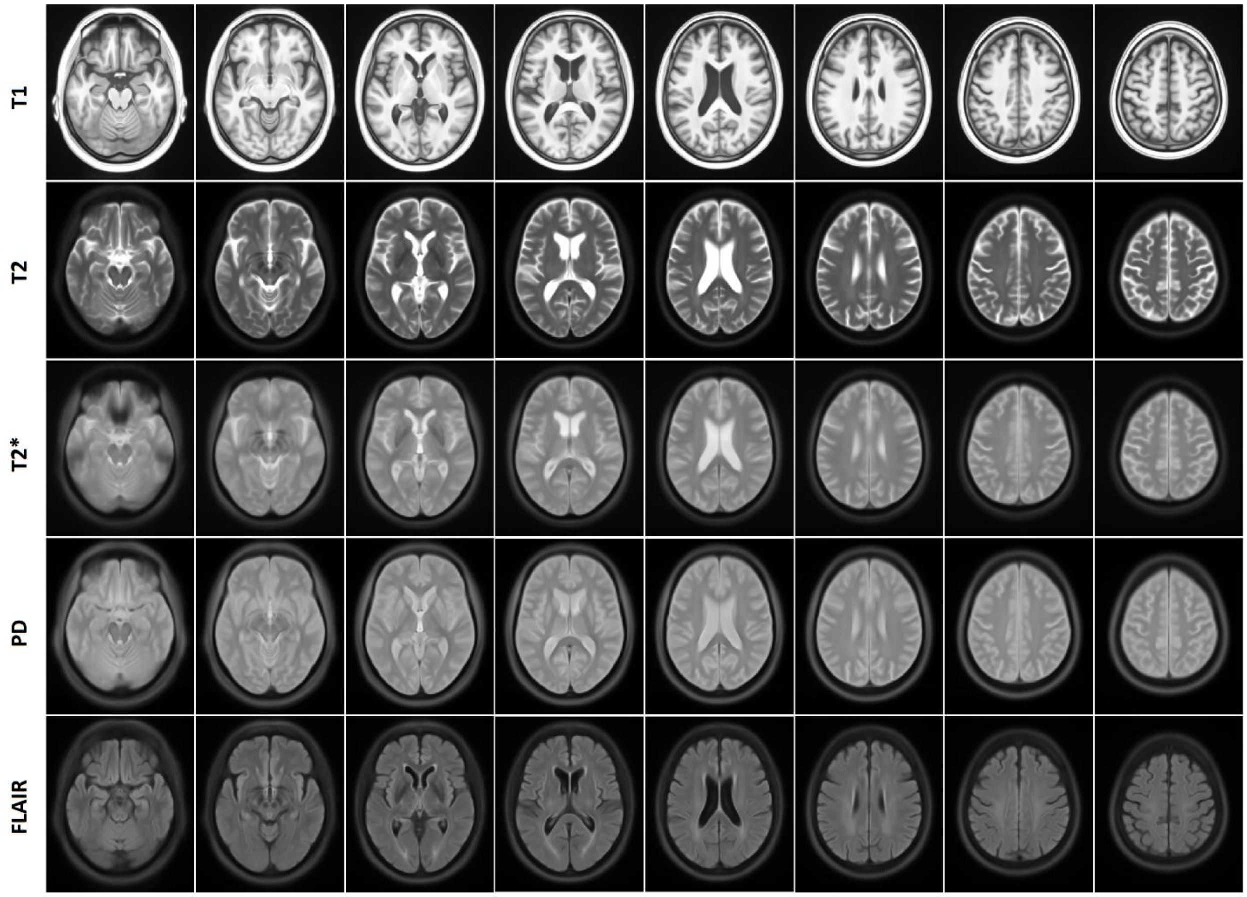

Figure S.19. Axial slices of average Male MCI templates for T1, T2, T2*, PD, and FLAIR sequences.

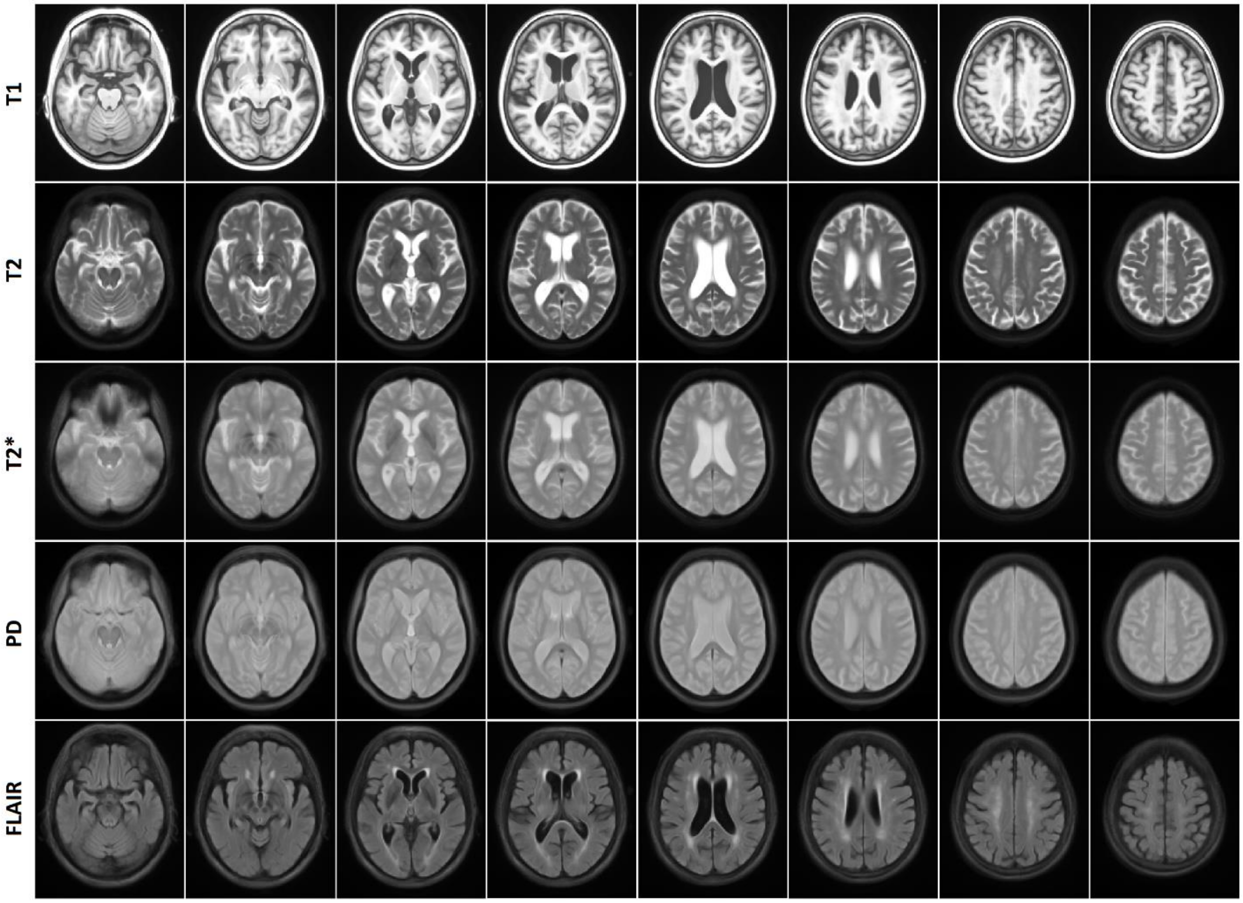

Figure S.20. Axial slices of average Female Mixed dementia templates for T1, T2, T2*, PD, and FLAIR sequences.

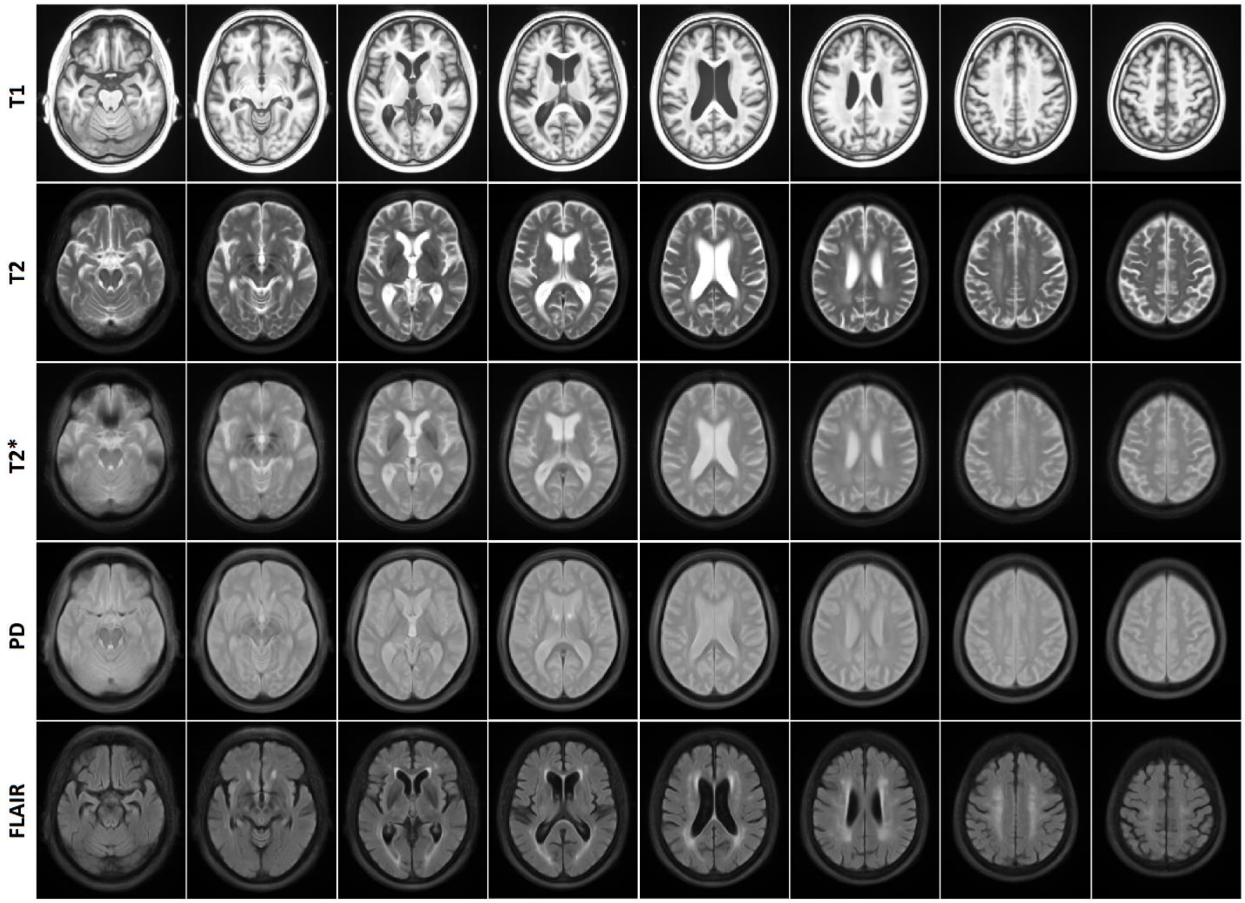

Figure S.21. Axial slices of average Male Mixed dementia templates for T1, T2, T2*, PD, and FLAIR sequences.

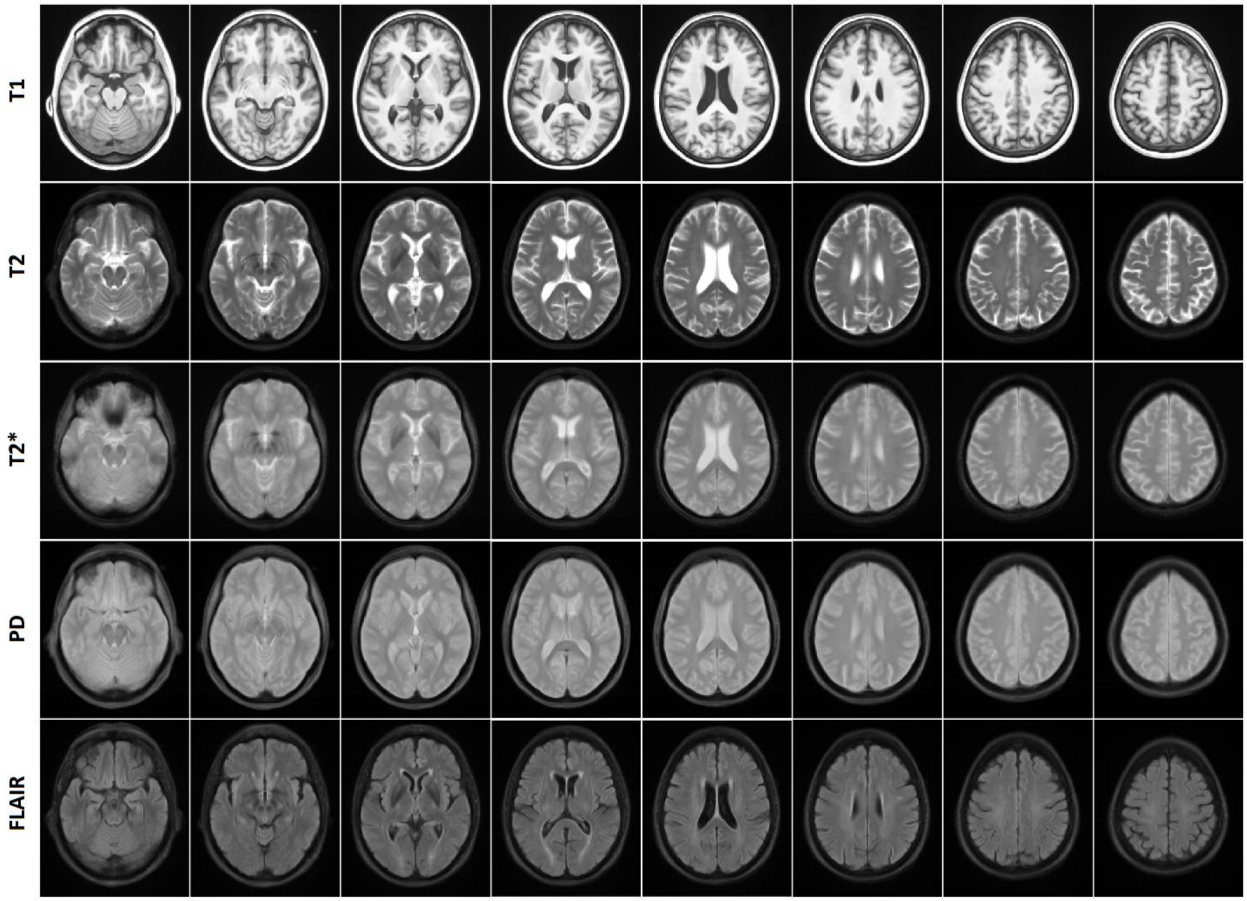

Figure S.22. Axial slices of average Female PD-CI templates for T1, T2, T2*, PD, and FLAIR sequences.

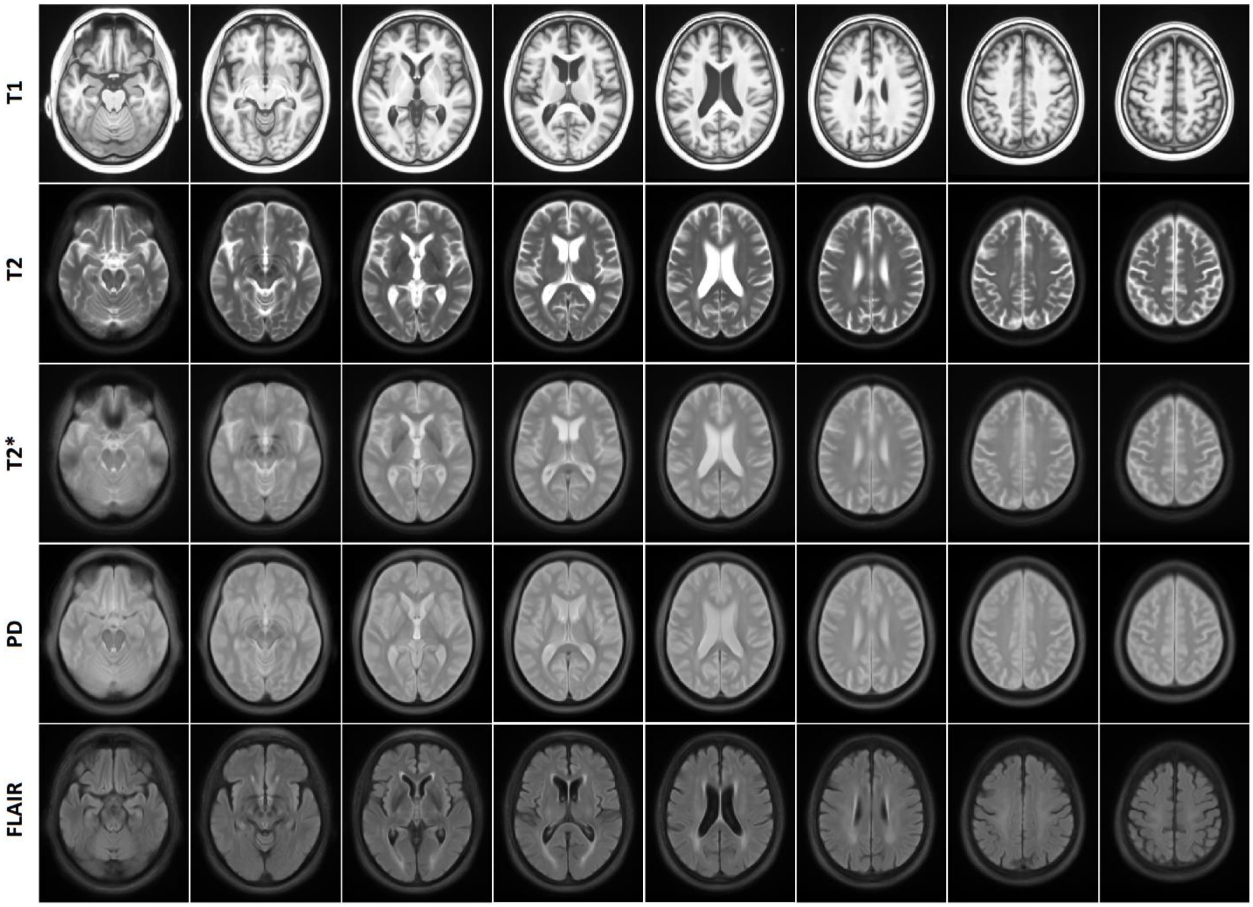

Figure S.23. Axial slices of average Male PD-CI templates for T1, T2, T2*, PD, and FLAIR sequences.

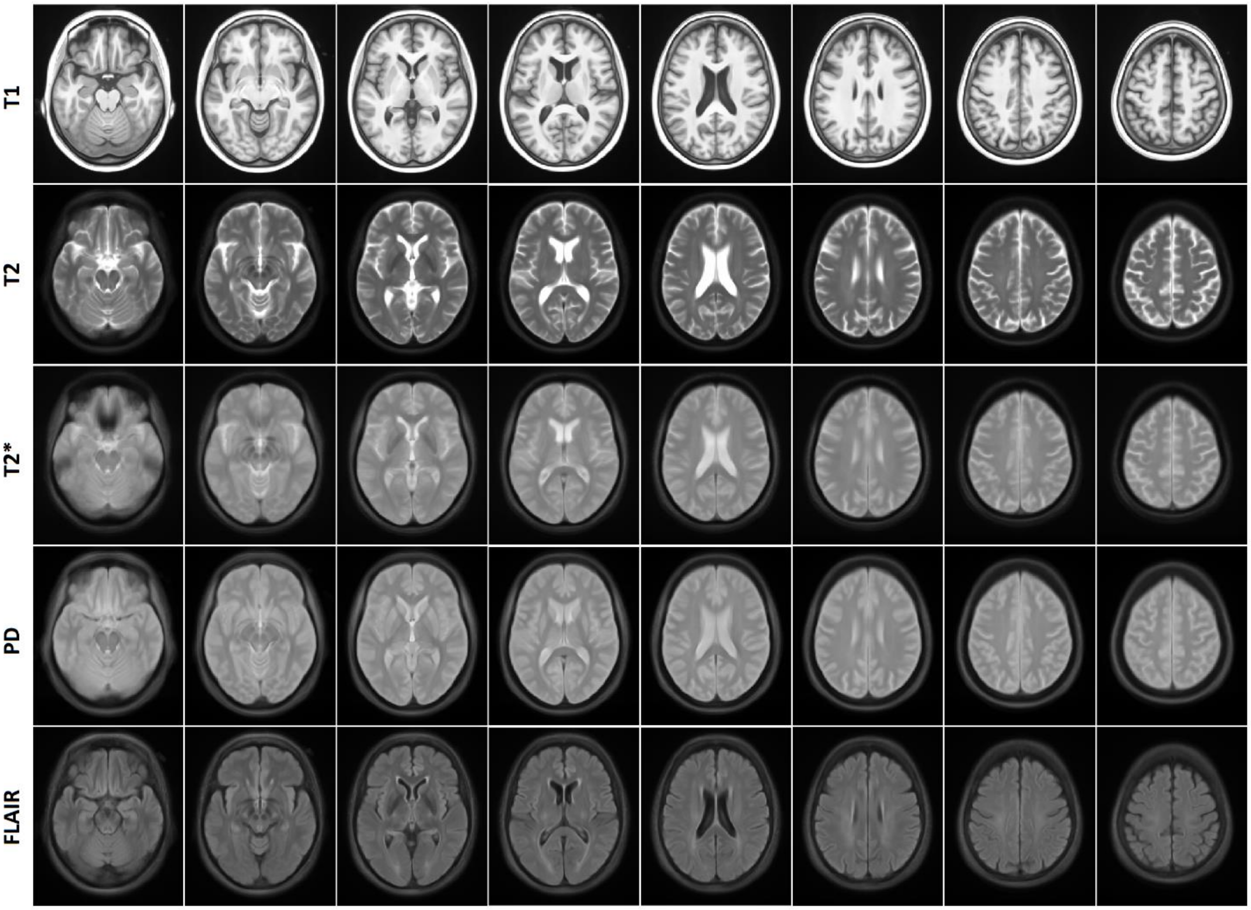

Figure S.24. Axial slices of average Female PD-CIE templates for T1, T2, T2*, PD, and FLAIR sequences.

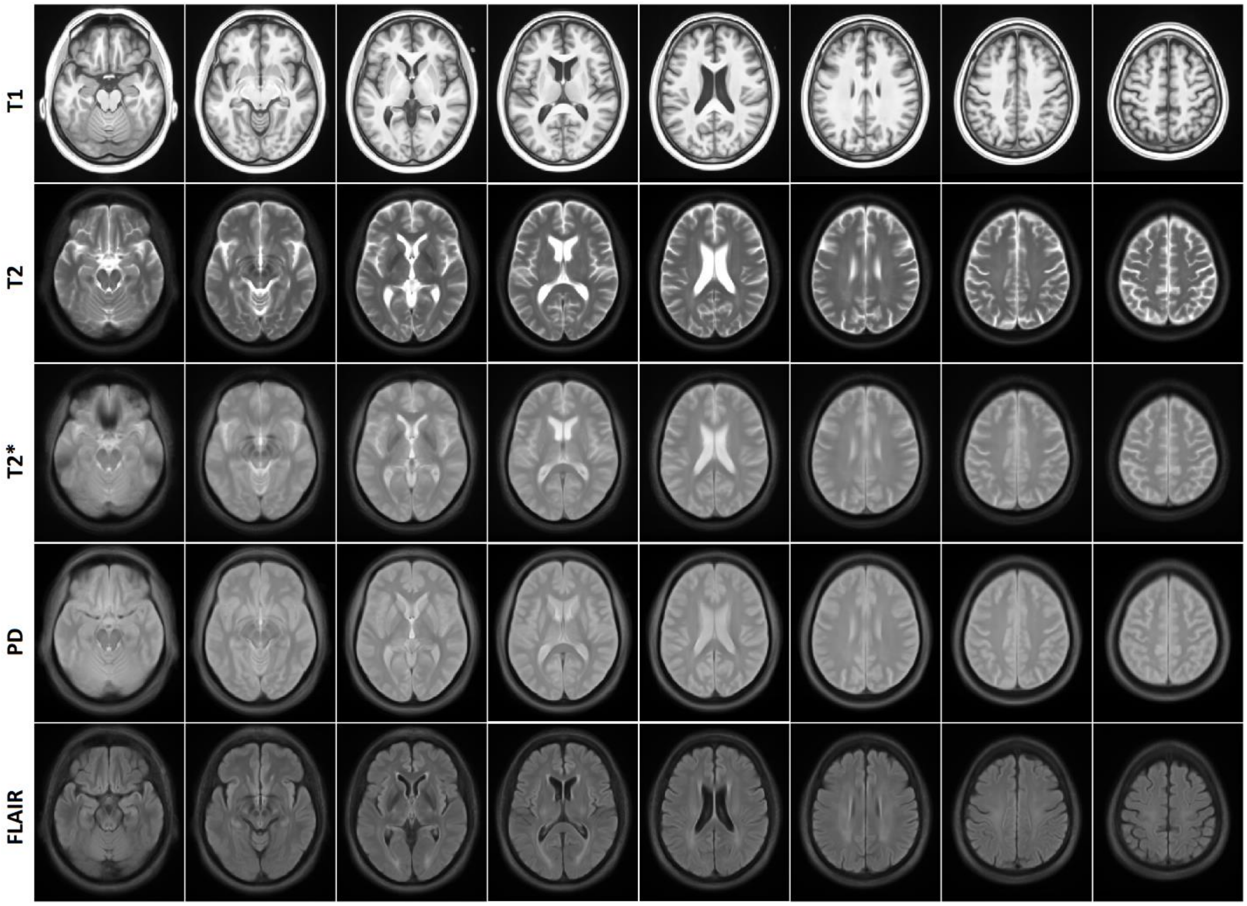

Figure S.25. Axial slices of average Male PD-CIE templates for T1, T2, T2*, PD, and FLAIR sequences.

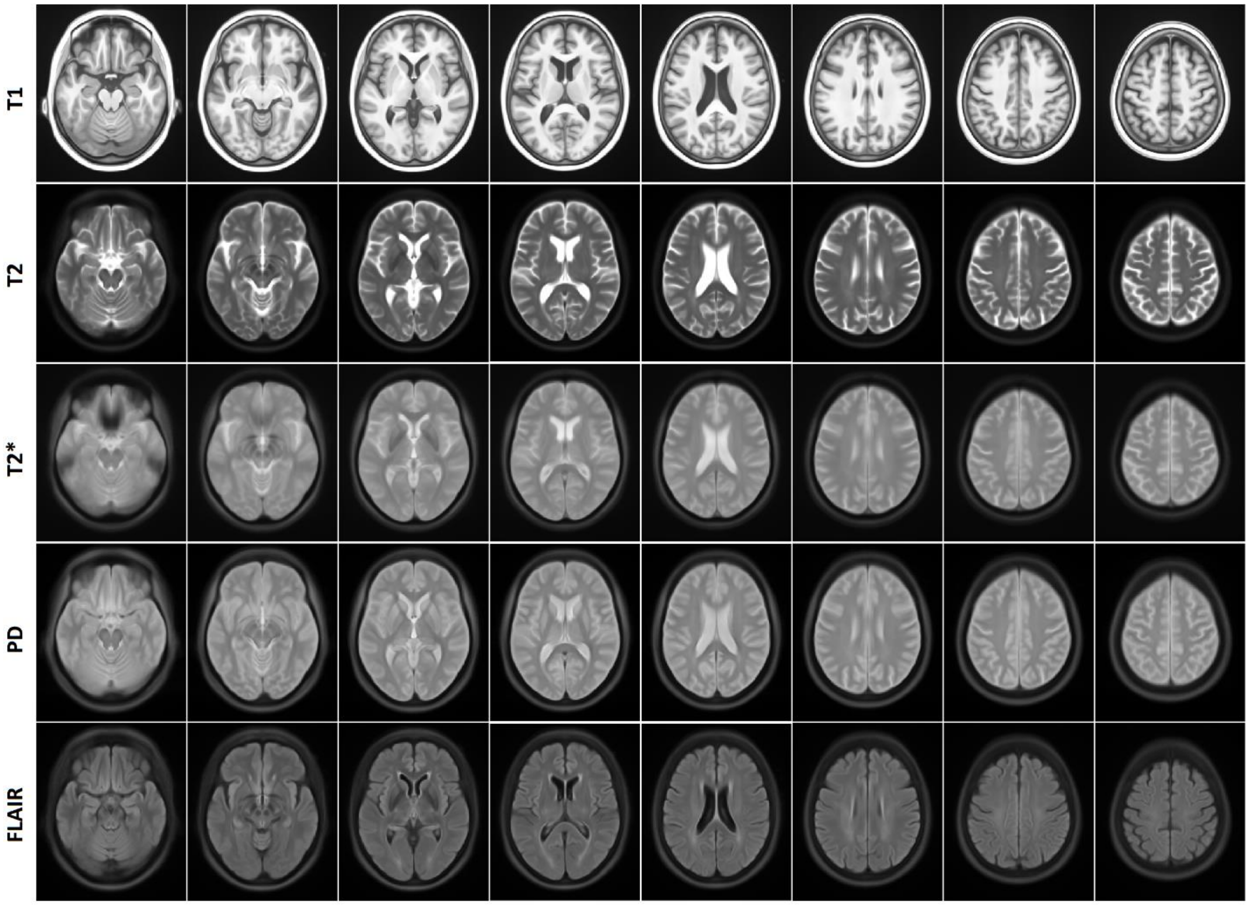

Figure S.26. Axial slices of average Female SCI templates for T1, T2, T2*, PD, and FLAIR sequences.

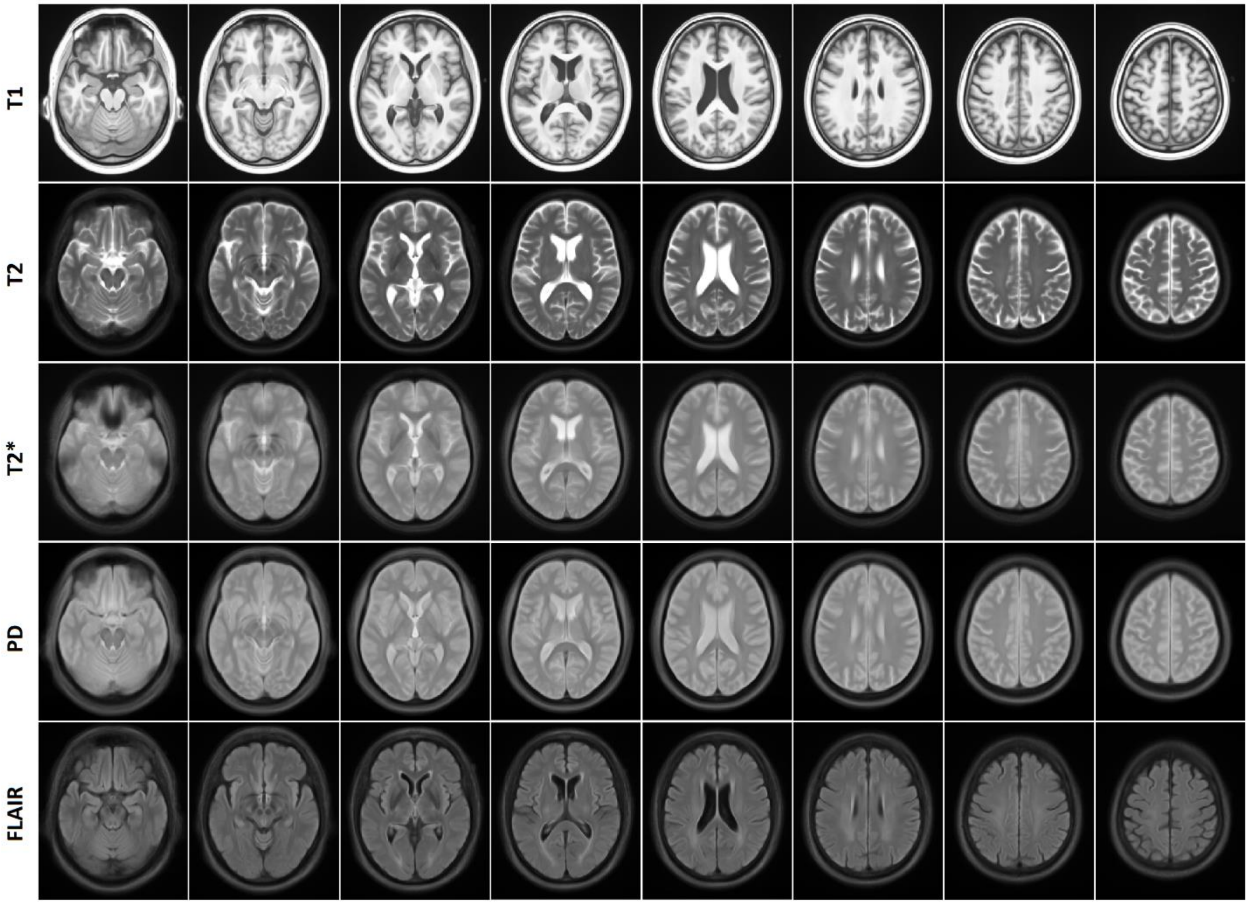

Figure S.27. Axial slices of average Male SCI templates for T1, T2, T2*, PD, and FLAIR sequences.

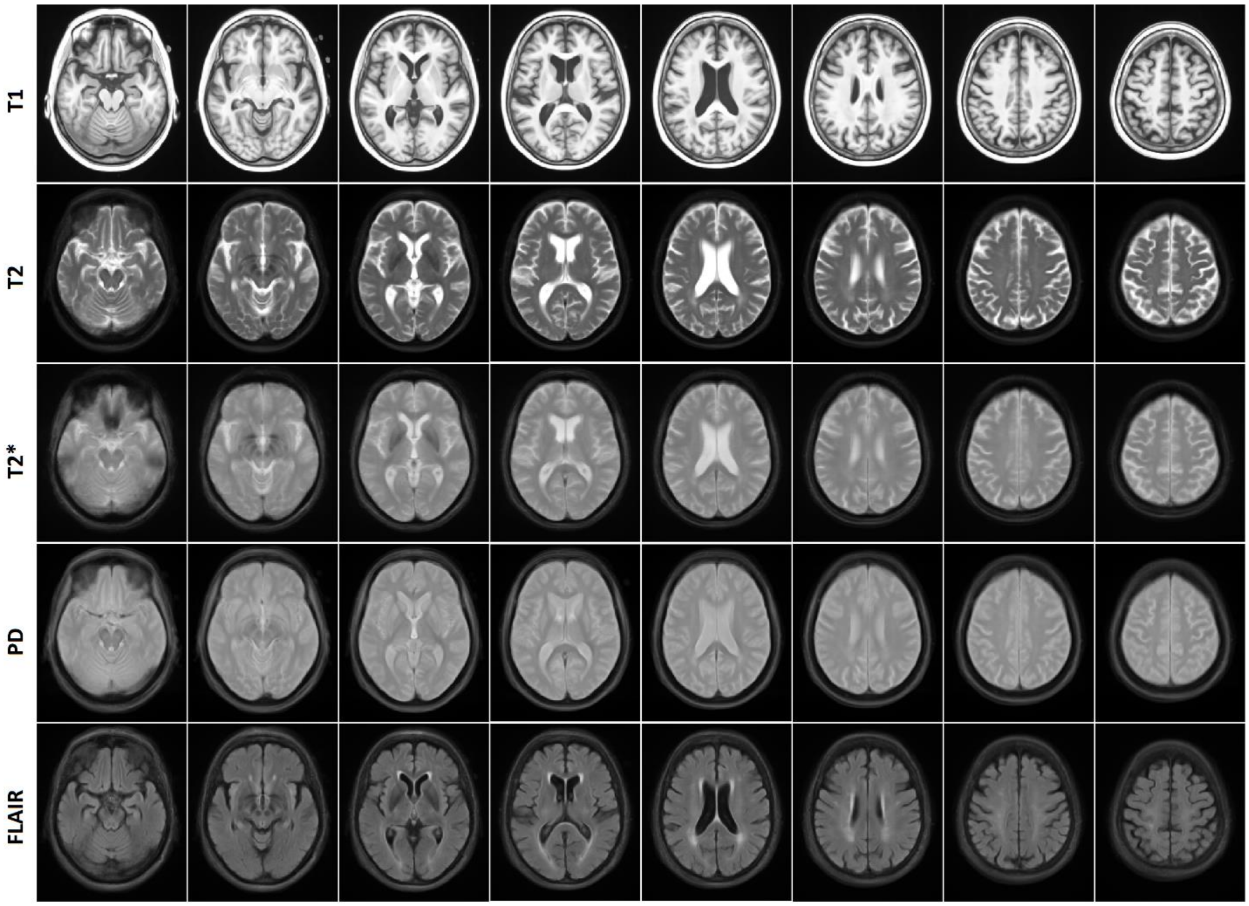

Figure S.28. Axial slices of average Female V-AD templates for T1, T2, T2*, PD, and FLAIR sequences.

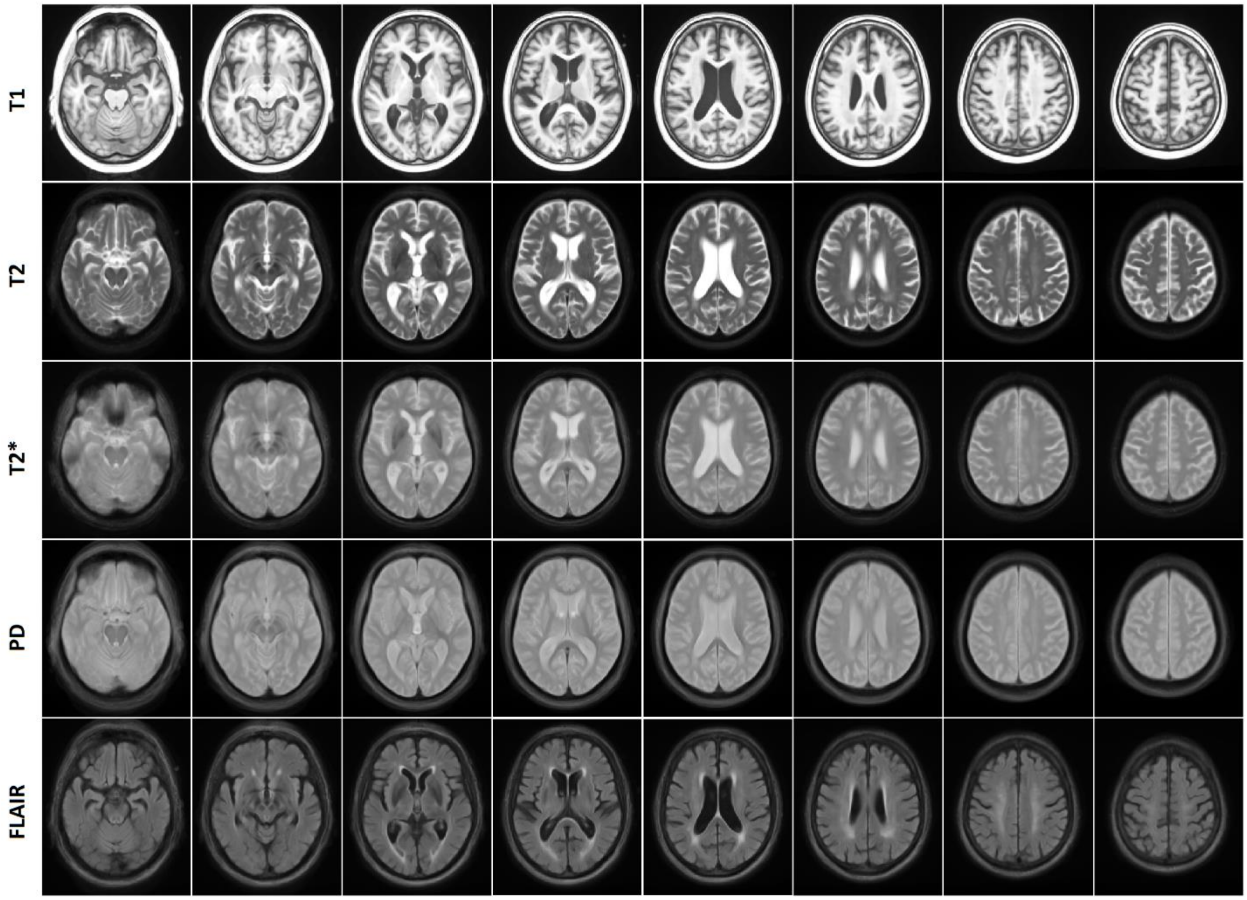

Figure S.29. Axial slices of average Male V-AD templates for T1, T2, T2*, PD, and FLAIR sequences.

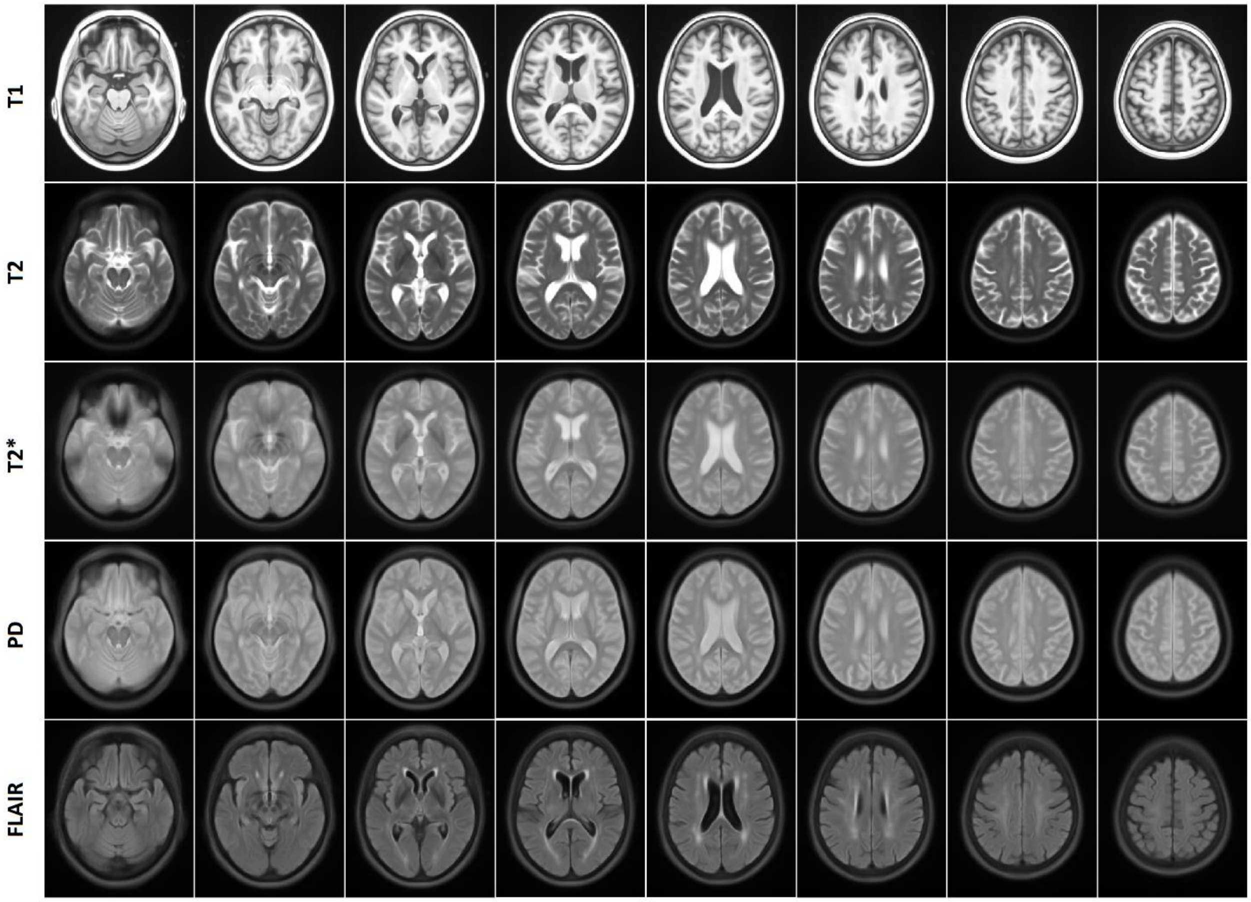

Figure S.30. Axial slices of average Female V-MCI templates for T1, T2, T2*, PD, and FLAIR sequences.

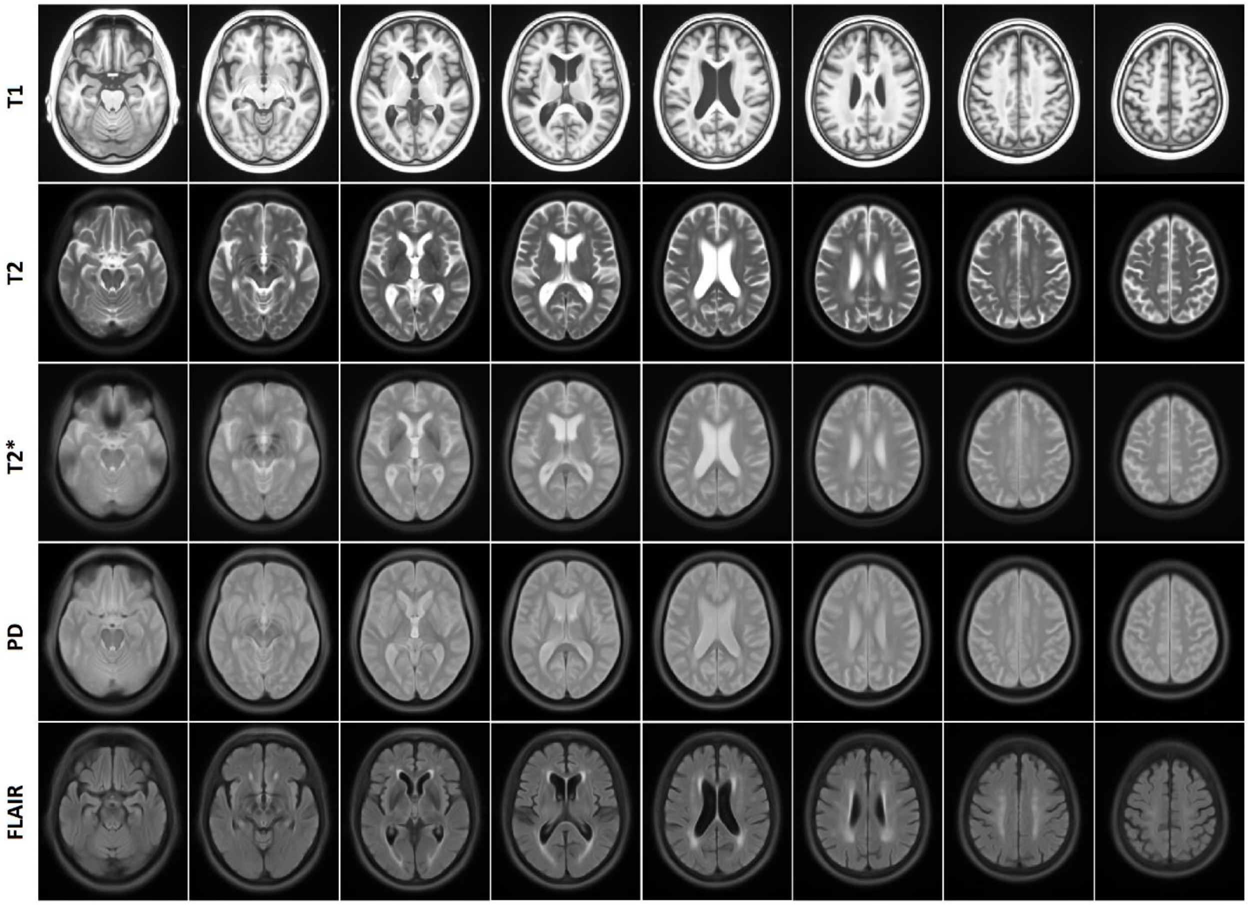

Figure S.31. Axial slices of average Male V-MCI templates for T1, T2, T2*, PD, and FLAIR sequences.
